# Glutamine alleviates the toxicity of externally applied amino acids in Arabidopsis

**DOI:** 10.3389/fpls.2026.1689741

**Published:** 2026-03-19

**Authors:** Lauren M. Kerwien, Rayah Edelson, Kristupas Vorobjovas, Guillaume Pilot

**Affiliations:** School of Plant and Environmental Sciences, Virginia Tech, Blacksburg, VA, United States

**Keywords:** amino acid metabolism, amino acid transport, ammonium, branched chain amino acids, nitrate, regulation - physiological, Target of Rapamycin (TOR)

## Abstract

**Introduction:**

Plants use amino acids not only as building blocks for protein synthesis, but also as nitrogen carriers between the various organs and as precursors of a plethora of specialized compounds that fulfill essential functions ranging from structural roles to interaction with other organisms. Feedback regulation of amino acid metabolic enzymes is well studied in plants and has been accepted as a main regulatory mechanism of pathway activity. Studies in *Nicotiana sylvestris* have shown that single amino acids provided at high concentrations are toxic to plants and plant cells, an inhibition that can be inexplicably reversed by addition of glutamine.

**Results and discussion:**

The present study established an Arabidopsis-based framework to further study the mechanisms controlling the activity of amino acid metabolic pathways. In this context, we determined with precision the toxicity of each amino acid on Arabidopsis plants grown in solid and liquid media. The level of toxicity varied between 0.3 and 4 mM, depending on the amino acid, with some amino acids found non-toxic at concentrations up to 20 mmol.l^-1^. For some of the tested amino acids, the inhibition was relieved by adding amino acids from the same pathway, supporting the notion that the effect is a consequence of the feedback inhibition of the pathway. In good agreement with past reports from *Nicotiana*, we found that glutamine alleviated, or sometimes completely suppressed, the growth inhibition exerted by single amino acids. Suppression of valine toxicity was not due to decreased valine uptake by glutamine, showing that glutamine effect is due to another phenomenon. The involvement of Target of Rapamycin (TOR) was tested using the specific inhibitor AZD8055. TOR inhibition completely overrode the toxicity of Val and masked the effects of Gln, in good agreement with TOR being a high order regulator of the metabolism, but precluding any conclusion as to whether it is involved in the Gln-suppressing effect of Val toxicity.

## 1 Introduction

Amino acids play a central role in plant metabolism, lying at the crossroads of the carbohydrate pathways and the nitrogen assimilation pathway. The only reaction by which nitrogen enters metabolism in physiological conditions is the synthesis of the amino acid Gln through the Glutamine synthetase/Glutamate synthase pathway; nitrogen is then incorporated into other nitrogenous compounds by transamination. Amino acids are used as substrates for many reactions leading to the synthesis of a plethora of secondary (specialized) metabolites essential for plant development, interaction with other organisms and for acclimation to growing conditions. Amino acids form a dynamic pool of metabolites whose distribution and content evolve over time depending on the conditions: during permissive conditions, amino acids can be stored in the vacuole (Winter et al., 1993; 1994) and used for protein synthesis, while they are degraded to provide energy or other metabolites during stresses (Huang and Jander, 2017; Hildebrandt, 2018).

Most amino acids are synthesized by several branched pathways, i.e. the Asp family (Lys, Thr and Met), the branched chain amino acid family (BCAA; Leu, Ile and Val), and the aromatic amino acid family (AAA; Trp, Tyr and Phe). The other amino acids are synthesized through more linear pathways (His, Pro, Ser, Gly, Cys) or by connected metabolic reactions (Glu, Gln, Asp, Asn, Arg, Pro, Ala). Most pathways are feedback-inhibited by their end products, namely amino acids, at the level of key enzymes forming complex regulatory loops (Pratelli and Pilot, 2014). Bioinformatic modeling using only this kind of information managed to accurately predict amino acid accumulation, suggesting that this form of regulation plays a major role in the regulation of the pathways, at least in steady state conditions (Curien et al., 2009). Yet, the activity of the metabolic enzymes is also subject to regulation at the post-translational level. For instance, Lys catabolism is regulated by phosphorylation (Miron et al., 1997; Zhu et al., 2002), and the synthesis of Leu is regulated by ubiquitin ligases (Iantcheva et al., 2022). Expression is also regulated at the mRNA level, with key enzymes of the pathways showing changes in their mRNA accumulation under various nutritional or stress conditions (e.g., Zhu-Shimoni and Galili, 1998; Stepansky et al., 2005; Less and Galili, 2008; Less et al., 2011). Regulatory cascades can even affect mRNA structure and stability, as in the case of the enzymes in the branch leading to Met synthesis (Hacham et al., 2006, 2007). These few studied cases hint at regulatory mechanisms acting at multiple levels and at the existence of broader regulatory pathways currently unknown.

Numerous studies have shown that several amino acids are toxic to plants at concentrations that varies from 100 µM to >10 mM, depending on the species, nature of the system (cells vs. whole plant) and medium (e.g., Borstlap, 1970; Gamborg, 1970; Behrend and Mateles, 1976; Bonner et al., 1996; Lee et al., 2007; Pratelli et al., 2010). Depending on the amino acid and its position in the biosynthetic pathways, the toxicity can typically be alleviated by supplying amino acids that are synthesized from the same pathway and take part into the regulation of the pathway; for instance Met relieves the toxicity of Lys+Thr, and Ile + Val relieves Leu toxicity (Dunham and Bryan, 1969; Borstlap, 1970; 1981). These observations are well in agreement with the fact that an amino acid in excess blocks the metabolic branch it controls, preventing the cells from synthesizing the other amino acids of the pathway and inducing an amino acid imbalance.

In apparent disagreement, Gln has been found to alleviate the toxicity of all amino acids (Bonner et al., 1992, 1996; Bonner and Jensen, 1997), while it has not been found to be a direct inhibitor/activator of any of the metabolic enzymes inhibited by the amino acid in excess. There, the authors postulated the existence of two kinds of inhibition: a general amino acid inhibition and a specific amino acid inhibition. The distinction comes from the ability of Gln to completely (general inhibition) or partially (specific inhibition) suppress the growth inhibition created by a single amino acid. The specific inhibition is thought to be created by certain amino acids (e.g. Val, Thr, Lys) that block biosynthesis of closely related amino acids in the same metabolic pathway, while the mechanism for the general inhibition is not clear. The authors hypothesized that inhibitory amino acids interfere with glutamine biosynthesis or exert osmotic effects opposite to Gln, like disrupting cell-volume regulation. In this case, Gln supplementation would restore the amino acid balance. Apart from its effect on growth inhibition by amino acids, Gln has been shown to alleviate plant’s response to the non-proteogenic amino acid β-aminobutyric acid (BABA). BABA induces a specific response that enhances resistance of Arabidopsis against microbial pathogens, but adding Gln to BABA-treated plants suppressed this response (Wu et al., 2010), suggesting that BABA triggers the above-mentioned general amino acid inhibition. A similar suppression effect of BABA-induced resistance to microbial pathogens by Gln has been recently shown in tomato (Janotik et al., 2022). Gln also has a role in promoting growth, both in *Nicotiana sylvestris* (Bonner et al., 1992) and Arabidopsis (Lardos et al., 2024), and promotes somatic embryogenesis and organ development in several species in presence of inorganic nitrogen sources (Vasudevan et al., 2004; Husin et al., 2014). A signaling role for Gln has been suggested (Lee et al., 2023), in good agreement with the fact that Gln is an activator of the Target of Rapamycin (TOR) regulator of metabolism (Cao et al., 2019; O’leary et al., 2020; Liu et al., 2021; O’leary et al., 2025). Other studies showed that Gln treatment of N-starved rice roots induced the expression of genes involved in nitrogen metabolism and in stress response (Kan et al., 2015). Growing Arabidopsis on Gln as nitrogen source also induced the expression of stress-responsive genes (Liao et al., 2024) and affected the expression of nitrate uptake transporters (Svietlova et al., 2024). It should be noted that these three last studies used Gln as the sole nitrogen source. The effects of Gln on plant metabolism thus appear complex and warrant more study.

In this context, the goal of this study was to establish reliable conditions for studying the regulation of amino acid metabolism in response to various stressors and molecules, determine the sensitivity levels of Arabidopsis to most proteogenic amino acids and test whether Gln can alleviate the growth inhibition caused by other amino acids.

## 2 Methods

### 2.1 Plant growth

Arabidopsis thaliana plants (Col-0) were surface sterilized in a 50% (v/v) ethanol/water 3% (w/v) sodium dichloroisocyanurate solution for 20 min on a shaker, washed three times in 100% ethanol, and the remaining ethanol was evaporated off under the sterile hood. Plants were grown on a base medium composed of half-strength Murashige and Skoog (MS) medium without nitrogen (Phytotech labs, M531) supplemented with the appropriate source of nitrogen (depending on the experiment) and sugar. The final concentrations of constituents were 0.5% (14.6 mM) sucrose, 1.5 mM CaCl_2_, 625 µM K_2_HPO_4_, 500 µM MgSO_4_, 50 µM MnSO4, 50 µM H_3_BO_3_, 50 µM Na_2_EDTA, 50 µM FeSO_4_, 15 µM ZnSO_4_, 2.5 µM KI, 0.5 µM Na_2_MoO_4_, 0.05 µM CoCl_2_, 0.05 µM CuSO_4_, 8 g/l agar if necessary, pH 5.7 KOH. Plants were grown in a growth chamber (Percival) set to 16/8 h day/night, 25/22 °C (day/night), with anti-condensation shelves.

For growth on solid medium, 12 seeds were sown in 9 cm-square Petri dishes filled with gelified base medium supplemented with 5 mM KNO_3_ and filter-sterilized amino acids (from 100 mM stock solutions stored at 4 °C, except Gln, Asn and Cys, which were stored frozen). Petri dishes were placed vertically, and plants were grown for 14 days in the growth chamber before scanning.

For growth in liquid medium, in the preliminary assays presented in **Supplementary Figures 2**, **3**, plants were sown directly in the growth medium (150 µl of a suspension of 1 mg/ml of seeds in 0.1% agar, which corresponds to 8 ± 2 seeds per well), treated 5 days later, and scanned and weighed after 7 more days (12 days total since sowing).

The newly optimized assay (in 48-well plates) consisted in germinating seeds on gelified base medium supplemented with 10 mM KNO_3_, and, after four days of growth, transferring three seedlings into each well of 48-well plates containing 900 µl of base medium supplemented, unless otherwise stated in figure legends or text, with 10 mM KNO_3_. At the time of transfer, the dry weight (DW) of one seedling was 46 µg ± 2 µg (n=5 pools of 20 plants), so that three plants weighed ~140 µg DW. Plants were treated 24 h later with the adequate volume of amino acids (from filter-sterilized 100 or 200 mM stock solutions) and water to a final volume of 1 ml. Plants were grown for nine days in the growth chamber, for a total of 14 days from sowing to harvest.

For some uptake assays, plants were sown in 1 ml of complete liquid half-strength MS medium (*i.e.*, 10 mM KNO_3_ and 20 mM NH_4_NO_3_ final) containing 1% sucrose, in a 24-well plate; the medium was renewed 10 days later. Plants were treated 13 days after sowing and the uptake measured after 24 h. For other assays, seeds were germinated on base medium supplemented with 10 mM KNO_3_ for four days, and six seedlings were transferred into wells of 24-well plates containing 2 ml of base medium supplemented with 10 mM KNO_3_. Seven days later, the medium was aspirated and replaced with 2 ml of fresh medium containing the appropriate amino acids. The uptake was measured 24 or 48 h after the treatment (see below).

### 2.2 Uptake assays

Plants were acclimated to the laboratory where uptakes were performed for about 1 h on a horizontally rotating shaker (Eppendorf Thermomixer set at 350 rpm). The growth medium was removed and replaced by 1 ml of the uptake solution composed of the same medium as for the growth, the desired unlabeled amino acids, and 1 µl of the ^3^H-labeled amino acid (37 kBq). The plants were incubated in the substrate for 15 min, washed three times with 5 ml 0.4 mM CaSO_4_ using a vacuum filtering device, and placed in 1 ml of the same medium as for the growth in liquid conditions for 15 min for efflux measurement. These two incubations occurred under shaking as above. The plants were dried for 3 hours at 60 °C and weighed, bleached in 1 ml 3% NaClO overnight, neutralized with 200 µl 1 M HCl, and ventilated for a few hours. Four ml of Optiphase HiSafe 3 scintillation fluid (Revvity) were added to the efflux medium and to the bleached plant mixture, and the radioactivity was measured in a liquid scintillation counter. Total uptake was calculated as the sum of the radioactivity in the efflux medium and the plants, divided by the plant dry weight. Note: the results of the efflux analyses are not reported here.

### 2.3 Data analysis

Data was graphed in Excel and JMP 18 and statistical analyses were performed in JMP or R. Normality of model residuals and homogeneity of variances were assessed using Shapiro-Wilk and Levene’s tests, respectively. Because ANOVA is robust to modest deviations from normality under approximately equal variances, Tukey’s HSD was used for *post hoc* mean separation. For amino acids where residual normality was not supported, results were verified using Welch’s one-way ANOVA, which produced consistent results. Dose response curves were created in JMP by fitting data points with 4-parameter logistic or 3-parameter exponential equations, from 8 concentrations of amino acids (including 0 mM), and three biological replicates (i.e. 3 wells). The inflection point of the dose response curve was used to calculate the inhibitory concentration 50% (IC50) for the logistic-fitted curves. For the exponential-fitter curves, IC50 was calculated using the inverse of the equation and the weight of plants halfway between the weight at the lowest and highest concentrations of each amino acid tested. A mixed model was used in JMP to analyze uptake data across multiple experiments, and the resulting adjusted data (obtained by subtracting the Best Linear Unbiased Predictor values to the observations) were used for the plots.

## 3 Results

### 3.1 Most amino acids are toxic to Arabidopsis roots

Previous amino acid toxicity experiments performed by our laboratory used half-strength standard MS medium with 0.5% sucrose (Pratelli et al., 2010). We reckoned that the presence of 10 mM ammonium in the medium (Murashige and Skoog, 1962) might interfere with the plant’s response to externally supplied amino acids, because ammonium is not typically stored in plant cells but readily converted to Gln. We also reduced the concentration of nitrogen similar to that of other publications (e.g. Lardos et al., 2024), to a concentration of 5 mM nitrate, supplied as potassium nitrate.

We chose the concentrations of amino acids by reference to our past results (Pratelli et al., 2010). We did not use Cys, Tyr and Trp because of their low solubility or instability. As before, Gln, Asn, Glu, Asp, Pro and Ala did not inhibit root growth when supplied at 20 or 10 mmol.l^-1^ (**Supplementary Figure 1**). Root architecture seemed affected by Glu, Asp, Pro and Ala but was not explored further(**Supplementary Figure 1**). The other amino acids, including the non-proteogenic amino acids ornithine (Orn) andcitrulline (Cit), were found to be toxic at concentrations ranging from 0.5 mM to 10 mM (**Supplementary Figure 1**). Cit and Orn toxicity can be explained by their metabolic connection to the Arg pathway, which is toxic (see diagrams in Pratelli and Pilot, 2014). It should be noted that we did not attempt here to identify the minimum concentration of each amino acid that inhibited root growth, which might be lower than the ones we used.

### 3.2 Supplying amino acids from the same pathway relieved toxicity of some amino acids

**Figure 1:**
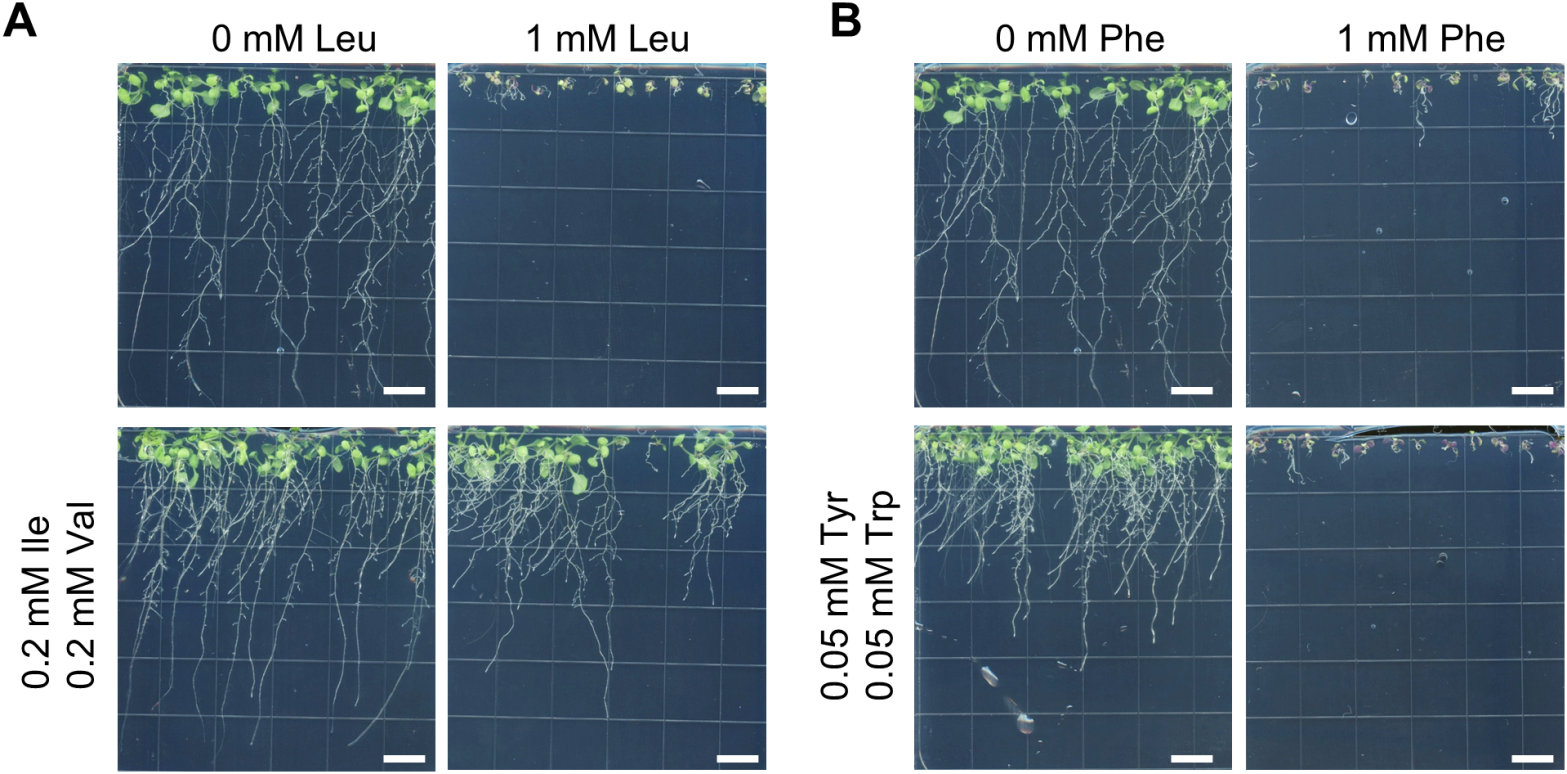
Recovery of the growth inhibition of Leu and Phe by amino acids from the same pathways. Inhibition of growth of Arabidopsis roots on media containing Leu **(A)** or Phe **(B)**, and recovery by Ile + Val or Tyr + Trp. Plants were grown vertically for 14 days on base medium containing 5 mM KNO_3_ and amino acids at the indicated concentrations. Scale bars = 1 cm.

It has been reported that, for the amino acids of the BCAA pathway, growth inhibition of *Nicotiana sylvestris* cells and *Spirodela polyrhiza* fronds by one amino acid can be suppressed by concomitant addition of the other BCAAs (Borstlap, 1981; Bonner and Jensen, 1997). To test whether this could also be observed with Arabidopsis roots, 0.2 mM of both Ile and Val were added to the medium containing 1 mM Leu. Almost complete suppression of the Leu inhibition was observed (**Figure 1A**). Interestingly, addition of 0.05 mM Tyr and Trp did not suppress the growth inhibition from 1 mM Phe, while they slightly reduced root growth when Phe was not present, proving that this concentration was biologically effective (**Figure 1B**).

### 3.3 Glutamine alleviates the root growth inhibition due to Leu and Phe

**Figure 2:**
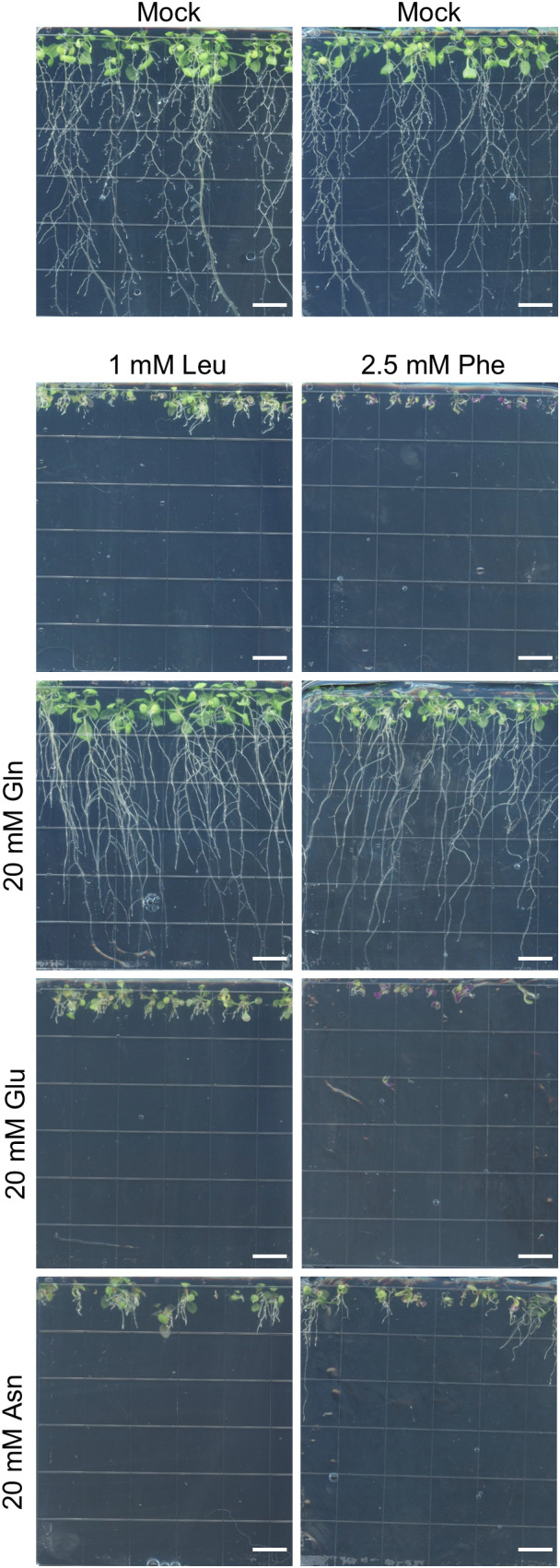
Recovery of the growth inhibition of Leu and Phe by Gln in a vertical plate assay. Plants were grown vertically for 14 days on base medium containing 5 mM KNO_3_ and amino acids at the indicated concentrations. Scale bars = 1 cm.

It was reported in *Nicotiana sylvestris* (Bonner et al., 1992, 1996; Bonner and Jensen, 1997), but never investigated in Arabidopsis, that Gln can suppress the growth inhibition created by other amino acids. This was tested for Leu and Phe, to which 20 mM Gln were added. In both cases, Gln alleviated the growth inhibition, leading to similar root growth as in the mock medium (**Figure 2**). It is conceivable that addition of a large excess of Gln to the medium would lead to a reduced uptake of Leu or Phe, thereby reducing their toxicity, as shown for Ala and Gly in *Spirodela polyrhiza* (Borstlap, 1974). Most amino acid transporters involved in amino acid uptake from Arabidopsis roots (mainly AtLHT1 and AtAAP1) characterized thus far have low substrate specificity and are therefore able to transport most amino acids (Rentsch et al., 2007). We thus reasoned that the Leu or Phe transport system should be similarly inhibited by a large excess of Glu (similar size as Gln, but different charge, or Asn, smaller than Gln but same charge). Transport through AtLHT1, an excellent Phe transporter, is indeed similarly competitively inhibited by Gln, Glu or Asn (Hirner et al., 2006), and AtAAP1 transports equally well Phe, Leu, Gln, Glu and Asn (Fischer et al., 2002). To test this hypothesis, Glu and Asn were added to the medium at the same concentration of Gln, but both amino acids failed to alleviate Leu or Phe inhibition (**Figure 2**). These experiments show that, similar to other plants and past reports, Gln can specifically alleviate growth inhibition by amino acids in this system.

### 3.4 Establishing growth conditions to study plant’s response to externally applied amino acids

Root growth is governed by the activity of the root apical meristem, which is subject to complex regulation involving hormones and the activity of TOR (Barrada et al., 2015; Ma et al., 2024). Studying the reason for the inhibition of growth by amino acids and its alleviation by Gln is difficult with vertically grown plants because of the low root tissue mass that Arabidopsis plants produce, the labor involved in sowing seeds on top of vertical agar dishes and the potential for contamination that renders one full dish not usable. Understanding the regulation of amino acid metabolism could reasonably be achieved if the whole plant is treated, without amino acids being first taken up by the roots, metabolized and translocated to the shoots. Such a system would also simplify material production and enable more conditions to be tested with more repetitions. While being more artificial than growth on gelified medium, we chose growing plants in wells of 48-well plates, completely submerged in medium: in this system, each well would correspond to a biological replicate, and many amino acid combinations and concentrations can be tested in parallel.

We first tested the effect of the amount (5, 10 or 20 mM) and nature of inorganic nitrogen (NH_4_^+^ or NO_3_^-^) on the tolerance of Arabidopsis growth to Leu and Phe. Whichever the nitrogen source,increasing amounts of inorganic nitrogen in the growth medium increased plant tolerance to both Leuand Phe toxicity (**Supplementary Figures 2**, **3**). Increasing percentages of ammonium also increased amino acid tolerance but led to areduction in the growth of mock-treated plants, especially when the medium contained 20 mM nitrogen(**Supplementary Figures 2**, **3**). The balance between growth of the control plants and amino acid susceptibility seemed to be attained in media containing 10 mM nitrogen.

We further explored the suitability of the medium composition, aiming at recapitulating pastobservations concerning root growth on vertical plates. We chose the *gdu1-1D* mutantas a positive control. This mutant over-expresses the *AtGDU1* gene, and displays enhanced amino acid export from plant cells, a concomitant decrease amino acid uptake and exceptional amino acid tolerance in half strength MS medium (Pratelli et al., 2010). *gdu1-1D* was grown alongside the wild type on media containing Leu or Phe with increasing percentage of ammonium, keeping the concentration of total inorganic nitrogen at 10 mM. For the wild type, growth difference between the control and amino acid treatments was maximal in 100% nitrate compared to the other media (**Supplementary Figure 3B**). While the growth of *gdu1-1D* plants was essentially the same betweencontrol and amino acid treatments on all media, their growth was reduced when the medium contained50% or more ammonium, suggesting that this mutant is ammonium sensitive, which has never been reported before. Because control plants appeared healthier when the medium contained 10 or 20 mM nitrate (**Supplementary Figure 2A**) and the plants were more susceptible to amino acids in media containing only nitrate (**Supplementary Figures 3A, B**), subsequent experiments were performed in media containing 10 mM nitrate as nitrogen source.

The final test for the suitability of this medium consisted in testing for the alleviation ofamino acid toxicity by Gln. Addition of 10 or 20 mM Gln, but not Glu, alleviated the toxicity of Pheand Leu supplied between 2 and 4 mM (**Supplementary Figure 4A**), in good agreement with the results of vertical plates (**Figure 2**).

### 3.5 Estimating the IC50 of toxic amino acids

**Figure 3:**
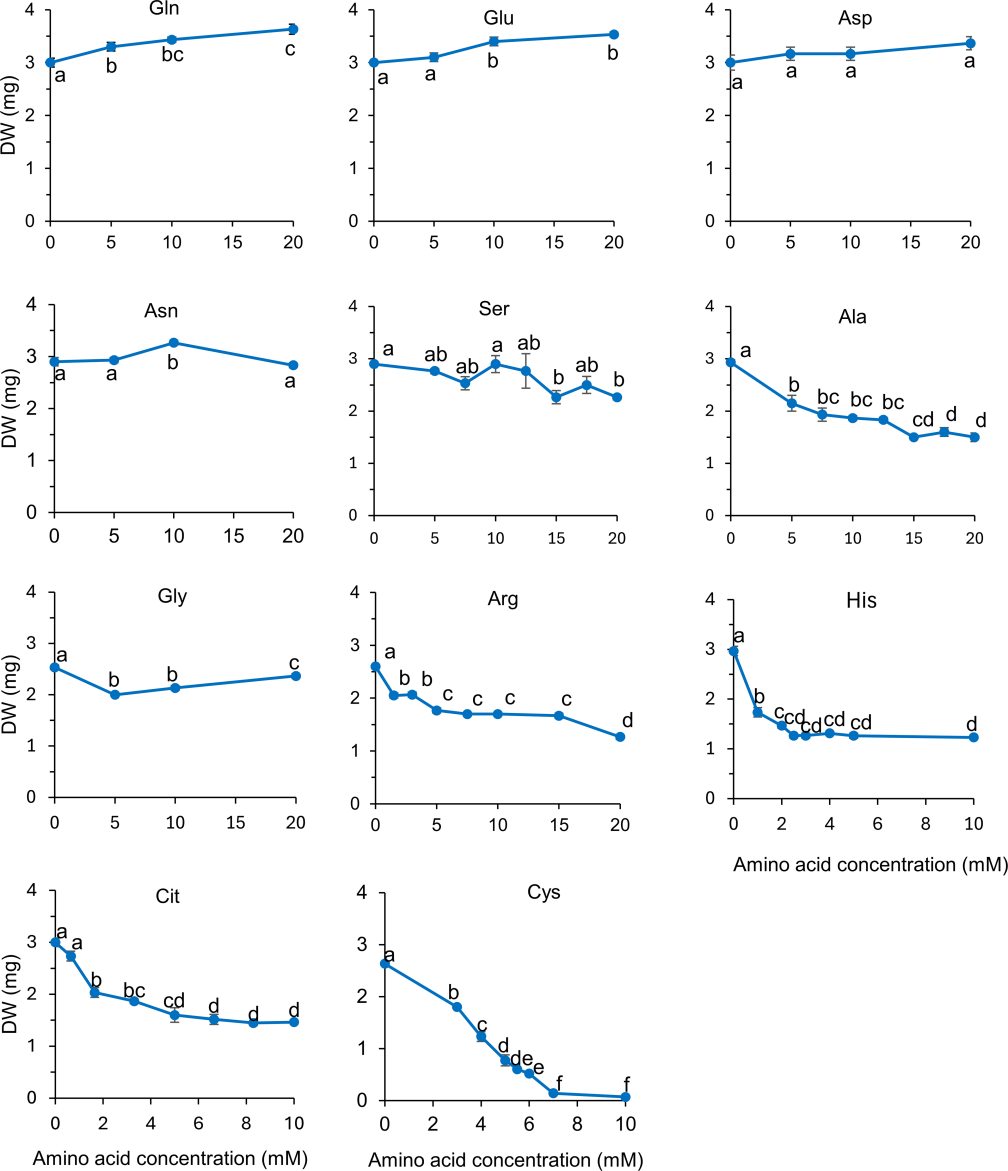
Dose response curves of the dry weight of Arabidopsis to various amino acids. After four days of germination on solid medium, plants were grown for 9 days in 48-well plates in liquid base medium containing 10 mM KNO_3_, supplemented with amino acids at the indicated concentrations (X-axes). For each amino acid, the effect of concentration on the dry weight was analyzed using a one-way ANOVA followed by a Tukey’s HSD, *P*-value <0.05. Error bars (when visible) represent the standard deviation from the mean, n=3. Different letters indicate statistically different means.

**Figure 4:**
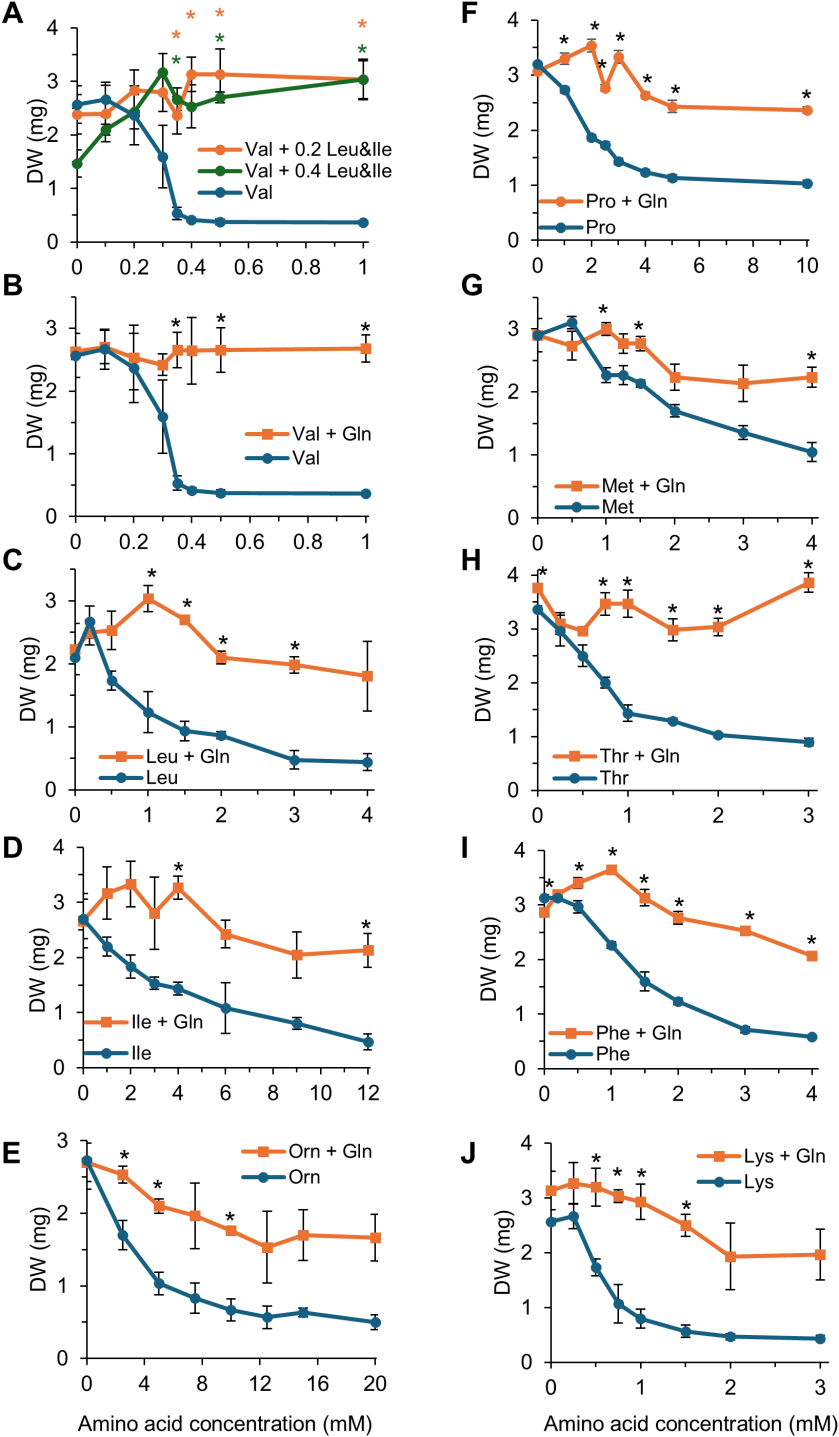
Gln alleviates the growth inhibition of all toxicity of most amino acids. After four days ofgermination on solid medium, plants were grown for 9 days in 48-well plates in liquid base mediumcontaining 10 mM KNO_3_, supplemented with amino acids at the indicated concentrations (X-axes). **(A)** Blue line: Val only; Green line: Val + 0.4 mM Leu + 0.4 mM Ile; Orange line: Val + 0.2 mM Leu + 0.2 mM Ile. At each amino acid concentration, dry weight was compared between plants grown in Val alone and plants grown in Val supplemented with either 0.2 or 0.4 mM Ile + Leu using a Welch’s t-test with a Holm-Bonferroni correction, p<0.05, n=3. An orange asterisk indicates a significant difference in plant growth when plants were grown in Val medium supplemented with 0.2 mM Ile + Leu compared to the Val medium. A green asterisk indicates a significant difference in plant growth when plants were grown in Val medium supplemented with 0.4 mM Ile + Leu compared to the Val medium. **(B-J)** Blue: amino acid only; Orange: amino acid + 20 mM Gln. * Significantly different from the growth in a medium containing only the toxic amino acid at the same concentration, Welch’s t-test with a Holm-Bonferroni correction, *P* < 0.05, n=3. Error bars (when visible) represent standard deviation from the mean. The pictures of the plants used to generate these data are presented in **Supplementary Figure 6**.

The toxicity of Arabidopsis wild type plants to amino acids was tested using the newly defined protocol involving transferring 4-days-old seedling from solid to liquid medium prior to treatment (see Methods), recording plant dry weight and images of the plates. Plants were exposed to three to eight concentrations of each amino acid. For toxic amino acids, the concentrations were chosen based on the results of preliminary experiment in which growth was visually estimated, so that the final concentrations spanned the range across which the growth declined from non-inhibited to completely inhibited (not shown). Gln, Glu, Asn and Asp were not toxic to plants when supplied at concentrations up to 20 mM (we deliberately did not test higher concentrations) (**Figure 3**). Gly, Ser and Arg were moderately toxic, leading to ~10, 20, 50 and 50% reduction in seedling dry weight, respectively, when supplied at concentrations up to 20 mM (**Figure 3**). His, Cit, Pro and Ala reduced plant growth by ~50 to 65%, leading to IC50s of 0.70, 1.5, 1.5 and 4.1 mM respectively (**Table 1**, **Figure 3**). The other amino acid tested (Cys, Orn, Val, Leu, Ile, Met, Thr and Phe) decreased plant growth by at least 70% (**Table 1**, **Figures 3**, **4**). Val, Thr, Leu and Phe were the most toxic with IC50 of ~1 mM or less in our conditions (**Table 1**). Interestingly, most of the toxic amino acids corresponded to the free amino acids typically less abundant in plants (“minor” amino acids), whose contents have been shown to be highly coordinated, even across species and conditions (Noctor et al., 2002).

**Table 1:** IC50 (mM) for AZD8055 and the tested amino acids on the growth of Arabidopsis grown for 14 days in liquid medium.

Compound	Exp1	Exp2	Exp3	Average
AZD	0.024	0.023		.0023
Val	0.31	0.30		0.31
Thr	0.38	0.49		0.44
His	0.56 *	0.46 *		0.51
Leu	0.98	0.60		0.79
Phe	1.25	0.84	1.20	1.10
Met	1.49	1.36		1.42
Pro	1.68 *	1.16 *	1.46 *	1.43
Lys	2.61 *	0.84 *		1.73
Cit	1.47 *	2.03 *		1.75
Ile	3.03 *	3.03 *		3.03
Orn	3.24	4.11 *	2.54 *	3.30
Cys	3.74	4.21	3.47	3.80
Ala	4.69 *	3.53 *		4.11
Arg				n.d.
Ser				Not toxic
Gly				Not toxic
Asp				Not toxic
Asn				Not toxic
Glu				Not toxic
Gln				Not toxic
Trp				n.a.
Tyr				n.a.

Results from two or three independent experiments (Exp1, Exp2, Exp3). Dose response curves were fitted with logistic equations, except for the experiments indicated with a star “*”, which were fitted with an exponential equation. IC50 could not be determined for weakly and not toxic amino acids (Gln, Glu, Asn, Asp, Ser, Gly and Arg). Trp and Tyr were not tested because of poor solubility. “n.d.” not determined; “n.a.” not assayed; “Not toxic” no effect on growth was observed for concentrations up to 20 mmol.l^-1^.

### 3.6 Effect of Gln or complementary amino acids on the toxicity of specific amino acids

Similar to the effect of Leu on root growth on vertical plates (**Figure 1A**), the toxicity of Val was completely relieved by addition of the two amino acids synthesized from the same pathway, Leu and Ile, supplied at 0.2 or 0.4 mM (**Figure 4A**). Interestingly, addition of 0.4 mM each of Leu and Ile was toxic to plants, with areduction of ~40% in dry weight, possibly because Leu and Ile at this concentration (but not at 0.2mM) prevents sufficient Val synthesis by feedback inhibiting the acetohydroxy acid synthase (AHAS, see diagram on **Supplementary Figure 5**).

The effect of Gln on the toxicity of Val, Leu, Ile, Orn, Lys, Thr, Met, Phe and Pro was tested across seven concentrations of each amino acid. For each amino acid, Gln significantly alleviated the growth reduction (**Figure 4**, **Supplementary Figure 6**). Complete suppression of toxicity was observed for Val, which was the amino acid with the lowest IC50, and Thr. Gln reduced, but did not completely suppress, the toxicity of the other amino acids, leading to plants that did not grow as much as the control.

**Figure 5:**
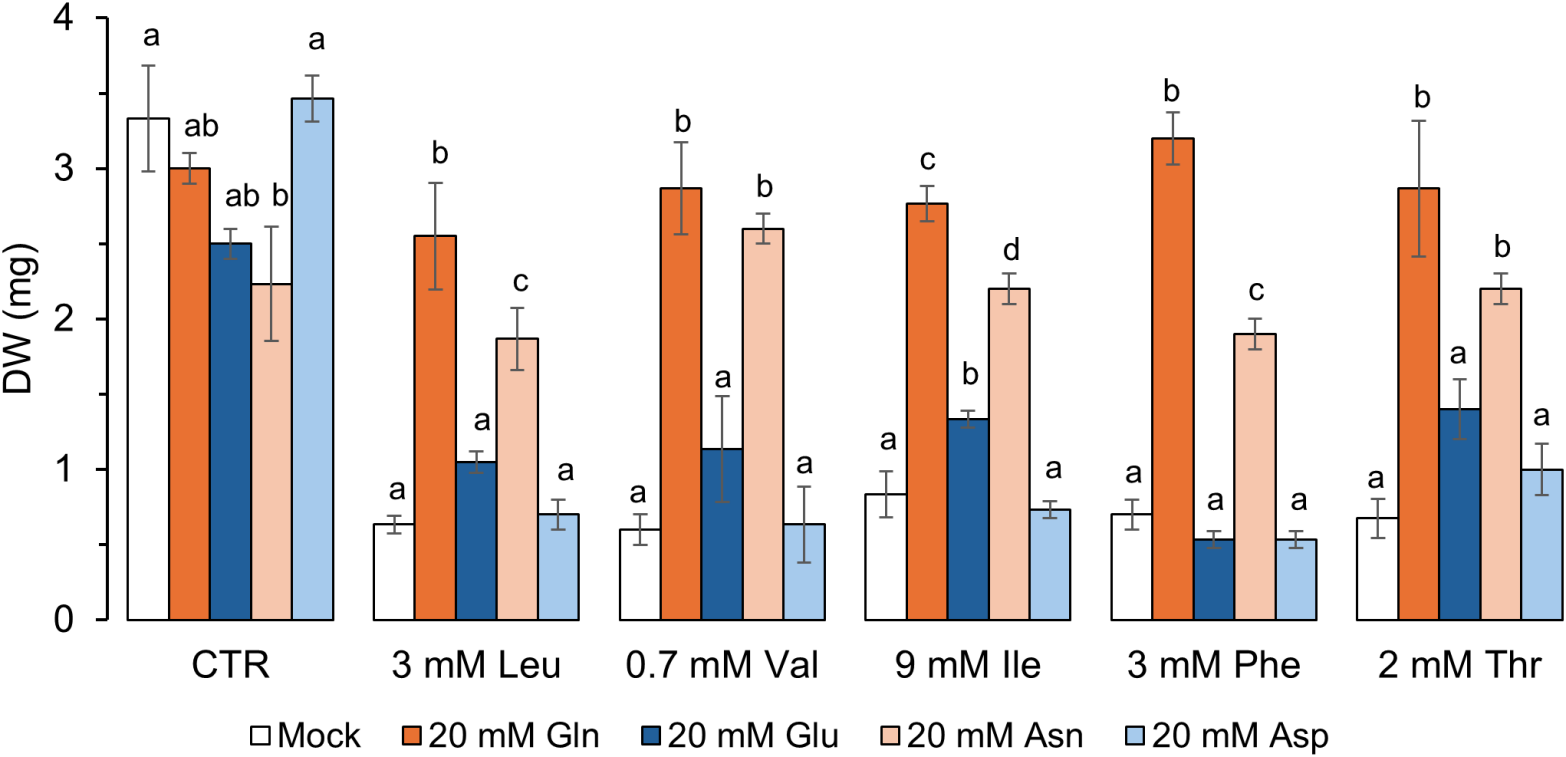
Gln and Asn alleviate the toxicity of Leu, Val, Ile, Phe and Thr in a liquid assay. After fourdays of germination on solid medium, plants were grown for 9 days in 48-well plates in liquid basemedium containing 10 mM KNO_3_, supplemented with amino acids (3 mM Leu, 0.7 mM Val, 9 mM Ile, 3 mM Phe, 2 mM Thr; CTR, no toxic amino acid) and 0 (mock) or 20 mM Gln, Glu, Asn and Asp. For each amino acid, the effect of concentration on the dry weight was analyzed using a one-way ANOVA followed by a Tukey’s HSD, *P*-value <0.05 (different letters indicate statistically different means). Error bars represent the standard deviation from the mean, n=3. The pictures of the plants used to generate these data are presented in **Supplementary Figure 4B**.

Because Asn and Asp were, with Gln and Glu, the amino acids that did not affect growth when supplied at concentrations lower up to 20 mmol.l^-1^, we tested whether they could alleviate the toxicity of other amino acids. We applied 20 mM Gln, Glu, Asp and Asn concomitantly with Leu, Val, Ile, Phe and Thr at concentrations well above their IC50. Both Glu and Asp failed to alleviate the toxicity, whereas Asn had a strong alleviating effect, yet not as much as that of Gln, on the toxicity of the five tested amino acids (**Figure 5**).

**Figure 6:**
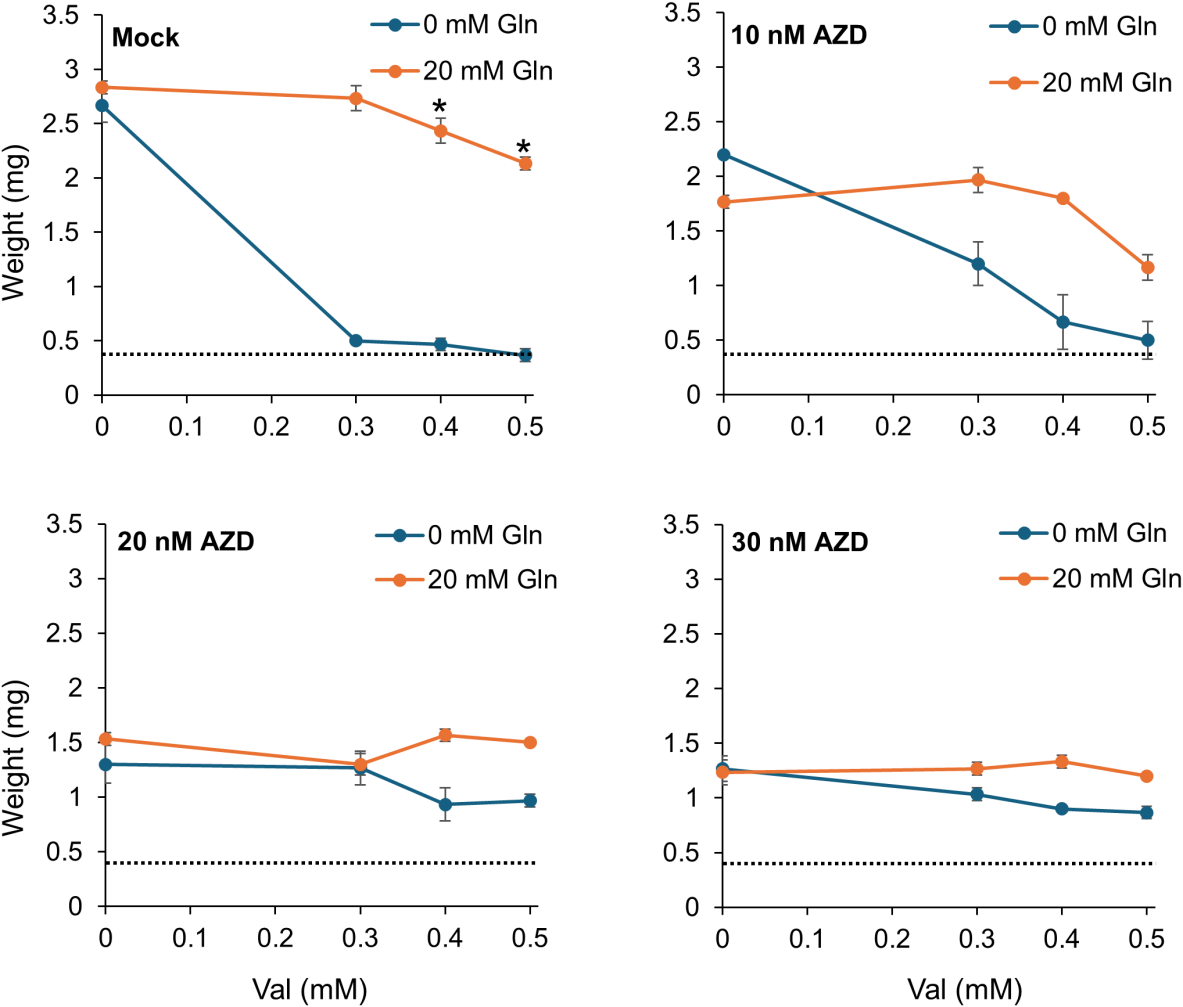
TOR inhibition overrides the effect of Val and Gln on plant growth in liquid medium. After four days of germination on solid medium, plants were grown for 9 days in 48-well plates in liquid base medium containing 10 mM KNO_3_, supplemented with 0, 0.3, 0.4 or 0.5 mM Val, with either 0 or 20 mM Gln and treated with 0, 10, 20 or 30 nM AZD8055 (AZD). Plant weights were compared between the Gln treatment at each Val concentration using a Welch’s t-test with a Holm correction, *P*-value<0.05, n=3. * Indicates a significant difference between plants supplemented with 0 and 20 mM Gln. There were no significant differences in plants treated with 0 or 20 mM Gln in the 10, 20, 30 nM AZD8055 treatments. The dotted line corresponds to the weight of plants treated with 0.5 mM Val only.

Gln has been shown to activate TOR (Liu et al., 2021; O’leary et al., 2025), which, among other effects, regulates amino acid metabolism. To test whether TOR is involved in the alleviation of amino acid toxicity by Gln, plants were treated by Val, Gln and AZD8055, a specific inhibitor of TOR (Montane and Menand, 2013). AZD8055 was supplied at concentration below, at and above its IC50 (i.e. 10, 20 and 30 nM; **Table 1**, **Supplementary Figure 7**). We reasoned that if AZD8055 and Val inhibit different pathways, their effect should be additive, and if Gln stimulates TOR to relieve Val growth inhibition, AZD8055 should suppress this effect. Unexpectedly, we observed that Val inhibition of growth (down to ~15% of the weight of the control) was suppressed by AZD8055 (to ~30% of the weight of the control), and that, at the same time, the relief of inhibition by Gln was suppressed by AZD8055 (**Figure 6**). In addition, the weight curves corresponding to the Val and Val+Gln treatments flattened with increasing AZD8055 concentrations to the point that they were nearly identical at 30 nM AZD8055, at a value corresponding to weights of plants treated with AZD8055 alone. These results suggest that inhibition of TOR overrides both Val toxicity and its relief by Gln in a concentration dependent manner.

### 3.7 Amino acid uptake is modified by the composition of the medium in response to externally supplied amino acids

**Figure 7:**
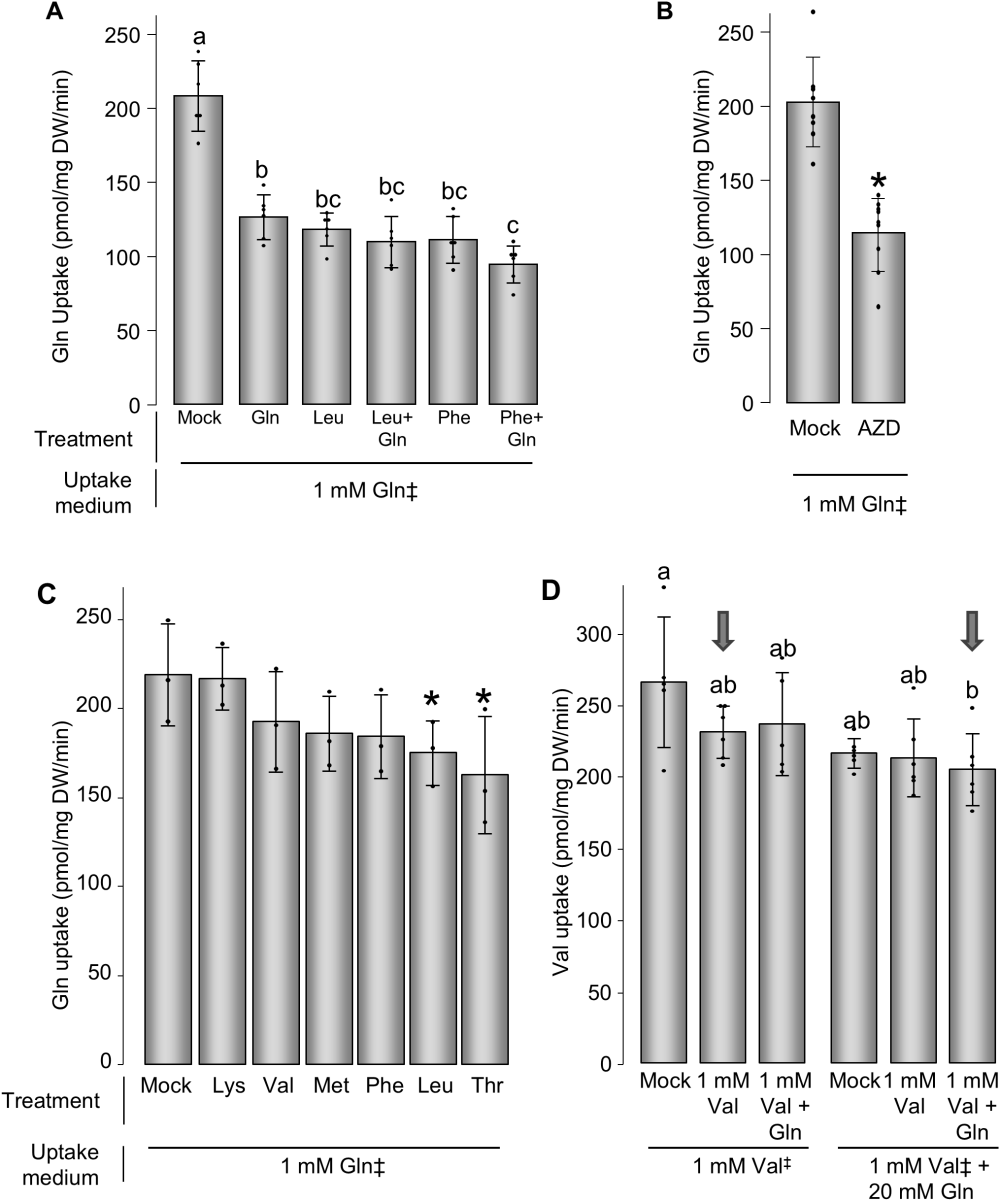
Amino acid uptake in Arabidopsis plantlets. **(A, B)** Plants were grown for 13 days in half-strength MS medium, treated by combination of amino acids provided at 10 mM each, or treated by 10 µM AZD8055 (AZD). The uptake of Gln (1 mM in the uptake medium) was assayed 24 h later. The bars and error bars are mean and standard deviation of 6 to 9 samples. **(C)** Plants were germinated on solid medium for four days and grown for 11 days in liquid base medium containing 10 mM KNO3, treated by amino acids provided at 10 mM each for 24 h, and the uptake of Gln (provided at 1 mM) was assayed. **(D)** Plants were germinated for four days on solid medium and grown for 11 days in liquid base medium containing 10 mM KNO_3_, treated by combination of amino acids provided at 1 mM and Gln at 20 mM for 48 h, and the uptake of Val (provided at 1 mM with or without 20 mM) was assayed. The bars and error bars represent means and standard deviations of 3 samples. “‡” indicates the radiolabeled that assayed for uptake. For **(A, D)**, different letters represent significantly different means, ANOVA, Tuckey’s HSD, p<0.05. For **(B, C)**, * indicates significantly difference from the control, Student’s t-test, p<0.05. .

To better understand the effect of the supply of amino acids at toxic concentrations on the physiology of the plant, uptake of amino acid was measured first in conditions used in previous publications (Pratelli et al., 2010), i.e. in plants grown in half-strength MS medium. Gln was used as a substrate for estimating overall amino acid uptake activity, since it is not toxic to plants and most amino acid transporters are not very specific for a particular amino acid (Rentsch et al., 2007). Treatments with either 10 mM Gln, Leu or Phe led to a similar decrease in Gln uptake (~50%), and addition of 10 mM Gln to Leu or Phe had no effect on the uptake compared to Leu or Phe alone (**Figure 7A**). Because many amino acids, like Gln and Leu, have been shown to stimulate the TOR kinase (Liu et al., 2021), we wondered whether the amino acid-triggered reduction in amino acid uptake could be due to TOR activation. Unexpectedly, treatment of plants for 24 h with AZD8055 led to the opposite effect, namely a reduction in Gln uptake (by ~50%, **Figure 7B**), similarly to treatments by 10 mM Gln or Leu.

Uptake of Gln when plants were grown in the same medium as the vertical growth assays presented in **Figures 1**, **2**, i.e. 5 mM KNO_3_, was also measured. In this medium, treatment of plants for 24 h with 10 mM Lys, Val, Met, Phe, Leu or Thr did not have an effect as marked as when plants were grown in half-strength MS medium (which contains 10 mM NH_4_^+^ and 20 mM NO_3_^-^): only Thr and Leu led to a decrease of Gln uptake, by ~20-25% (**Figure 7C**).

### 3.8 Suppression of Val toxicity by Gln is not due to reduced Val uptake

One major hypothesis that could explain the suppression of Val toxicity by addition of a large excess of Gln (**Figure 4B**) would be that Gln competes with Val for uptake. While this hypothesis was not supported by previous experiments (see 3.1 and 3.6 by testing the effect of Glu), an effect specific of Gln but not of Asn or Glu could not be neglected. Plants were treated for 24 h by 1 mM Val or 1 mM Val + 20 mM Gln and Val uptake was measured in a medium containing either 1 mM Val or 1 mM Val + 20 mM Gln (**Figure 7D**). Addition of any combination of amino acids had no or little effect of the uptake of Val, in good agreement with the results presented in **Figure 7C**. Notably, Val uptake was identical when plants were treated and assayed in 1 mM Val and when they were treated and assayed in 1 mM Val + 20 mM Gln, conditions that perfectly mimic those from **Figure 4B**.

## 4 Discussion

### 4.1 Establishing a robust assay to study the regulation of amino acid metabolism

We developed a method to grow Arabidopsis plants in liquid medium and apply a range of treatments, with a short turnaround time of 14 days. Nitrogen and sucrose concentrations, as well as germination and treatment timings, were optimized to ensure reproducibility: IC_50_ values obtained from experiments performed several months apart were comparable, with increased variability observed for less toxic amino acids (**Table 1**). Because germination and early vegetative development are controlled by distinct regulatory pathways (see below), treatments applied at sowing inevitably combine effects on germination with effects on the cellular processes of interest. Applying treatments four days after sowing circumvents this limitation and enables specific analysis of vegetative cellular responses. In this study, assay output was based on dry weight measurements, but additional readouts, such as chlorophyll content, could also be obtained after extraction and direct measurement in the 48−well plate. The robustness of the culture system likely relies on treating a fixed number of plants (three) in a defined volume of medium (1 ml), which enables time−course experiments by harvesting plants at successive time points, or pooling wells to obtain sufficient material for metabolite, protein, or mRNA analyses.

### 4.2 Gln alleviates the toxicity of all tested amino acids in Arabidopsis

Similarly to previous studies (e.g., Borstlap, 1970; Gamborg, 1970; Behrend and Mateles, 1976; Bonner and Jensen, 1997; Lee et al., 2007; Pratelli et al., 2010), excess of specific amino acids in the growth medium inhibited whole plant development when grown in liquid conditions, or root elongation when grown on Petri dishes placed vertically (**Figures 3**, **4**; **Supplementary Figure 1**). Not all amino acids were found to be toxic, and the IC50 of the toxic amino acids varied between 0.5 and 4 mM, with Val, Thr and His being the most toxic, and Asp, Glu, Asn and Gln showing no toxicity at the assayed concentrations (**Table 1**). Growth inhibition by Leu or Val was alleviated by supplying amino acids that are synthesized from the same pathway and take part in the regulation of the pathway, i.e. Ile+Val or Leu+Ile (**Figures 1A**, **4A**). Yet, this was not observed for Phe when Trp+Tyr were supplied (**Figure 1B**). Similarly, we did not manage to alleviate Ile toxicity by supplying Leu+Val at concentrations varying from 0.1 to 0.6 mM, or trying amino acid ratios similar to that described for *Spirodela polyrhiza* (Borstlap, 1981; not shown). It is possible that we have not provided Arabidopsis-specific ratios of those complementary amino acids, and more exploration of this phenomenon for Ile, Phe and possibly Met and Lys should be performed to draw definitive conclusions. Overall, the toxicity of specific amino acids likely stems from their feedback−inhibitory effects on the corresponding biosynthetic pathways. However, the levels of ‘minor’ amino acids (those present at lower concentrations, i.e. His, Arg, Tyr, Trp, Phe, Met, Val, Ile, Leu, Lys) are highly coordinated in plants (Noctor et al., 2002; Foyer et al., 2003), likely reflecting uncharacterized regulatory mechanisms. It is conceivable that disrupting this balance by supplying exogenous amino acids could destabilize metabolism and translation, ultimately causing stress or reduced growth, although this remains speculative and warrants further investigation.

Gln has been shown to alleviate the growth inhibition from exogenously applied amino acids in *Nicotiana sylvestris* cells (Bonner et al., 1996). Here, we show that Gln also alleviates amino acid-mediated inhibition in Arabidopsis grown vertically or in liquid culture (**Figures 2**, **5**). Similar to the study in *N. sylvestris*, the alleviation of the inhibition was not always complete. It depended on the ratio of the toxic amino acid over Gln: a higher ratio was associated with a weaker alleviation, *e.g.* for Phe, Leu or Ile (**Figure 4**). At amino acid concentrations that did not completely inhibit growth, the alleviation was always complete. These results are slightly different from *N. sylvestris*, where Gln could not completely alleviate the inhibition of Ala, Cys, Pro, Thr and Val (Bonner et al., 1996). Other differences were also noted compared to the reports from Bonner et al., 1996: the authors reported a toxic effect of Asn, Asp and Glu, that we did not observe, whereas, in liquid medium, Asn was also potent at alleviating amino acid toxicity (**Figure 5**). Differences in medium composition (full strength MS medium, i.e. 40 mM NO_3_^-^ and 20 mM NH_4_^+^, vs. 10 mM NO_3_^-^) or the organism studied (*N. sylvestris* cells vs. Arabidopsis plantlets) could explain those discrepancies. Interestingly, we did not observe alleviation of toxicity by Asn in vertical plates, where the seeds are germinated on the amino acid medium, while it was observed in the liquid assay where plantlets are transferred after germination into such medium. Germination is controlled by numerous checkpoints and complex regulatory pathways involving phytohormones and reactive oxygen species ensuring that the external conditions are optimal for the establishment of the developing seedling (Xu et al., 2024; Zhao et al., 2024). Some amino acids could therefore trigger different signaling cascades and responses during or after germination, leading to them being toxic when the seeds are sown rather than when the seedlings are transferred into the amino acid medium.

Similar to the study on *N. sylvestris* cells (Bonner et al., 1996), and different from other studies (Borstlap, 1974; 1978), who showed that alleviation of Leu, Val and Ile toxicity by Glu, Gly and Ala was due to an inhibition of uptake by competition, alleviation of the uptake of Val was not inhibited by a 20-fold excess of Gln (**Figure 7D**). This suggests that Gln plays a specific role in the regulation of metabolism (see also Lee et al., 2023), independent of an effect on amino acid uptake. Testing whether the effect of Gln is generalizable to all plants and across many conditions will be of particular interest. Gln concentration varies greatly between day and night, sub-cellular compartments and tissue for most studied species (Riens et al., 1991; Winter et al., 1992, 1993; Farré et al., 2001), likely reflecting differences in nitrogen and carbon availability. This raises the question of whether such condition- and species-dependent fluctuations affect amino acid fluxes, e.g. promoting synthesis when carbon and nitrogen are plentiful, possibly by acting as a part of a regulatory mechanism that tunes nitrogen metabolic activity to the current physiological status of the cell.

### 4.3 The quality and quantity of the inorganic nitrogen source affects the toxicity of and uptake response to amino acids

The assays performed to develop conditions for a robust liquid assay of amino acid toxicityshowed that increasing the quantity of total inorganic nitrogen and/or ammonium decreased the growthinhibition for Leu and Phe (**Supplementary Figures 2**, **3**). It is important to note that we have not tested other amino acids, but since Leu and Phe are synthesized from two different pathways, we do not believe that this observation is amino acid or pathway specific. This hypothesis is supported by a previous report showing that presence of ammonium in the medium suppressed the toxicity of most amino acids to tobacco, tomato and carrot cells (Behrend and Mateles, 1976). Increase of nitrogen content and especially NH_4_^+^ in the medium increases production and accumulation of Gln in plant cells (Frechilla et al., 2002; Hachiya et al., 2021). Given that external Gln alleviates amino acid toxicity, it is conceivable that Gln endogenously synthesized under high nitrogen/ammonium supply has the same effect, suggesting that the alleviation does not (at least entirely) depend on sensing external Gln, but rather from an intra-cellular effect. Through a similar reasoning, Asn treatment could leads to increase of Gln accumulation in the cells, by first being deaminated by asparaginase, and the resulting ammonium being used to synthesize Gln (Ivanov et al., 2012; Gaufichon et al., 2016), ultimately alleviating amino acid toxicity. This hypothesis could by tested by comparing amino acid profiles in NH_4_^+^-, Asn- and Gln-treated plants, in controlled pH conditions limiting ammonium toxicity (Hachiya et al., 2021), and/or reduce Gln synthesis using methionine sulfoximine.

Aside from amino acid toxicity and its possible effect on Gln content and signaling, increasing nitrogen or ammonium content in the medium affected how the plants responded to amino acid supply. In a medium containing 10 mM NO_3_^-^, addition of 10 mM Leu, Phe, Met, Val, Lys or Thr had no effect on the amino acid uptake capacity, tested by measuring the uptake of Gln (**Figure 7C**), while in a medium containing 20 mM NH_4_^+^ and 40 mM NO_3_^-^, 10 mM Leu, Phe and Gln treatments decreased the uptake of Gln by about 40% (**Figure 7A**). Whether this effect is due to changes in internal concentrations of amino acids caused by different sources of external inorganic nitrogen will be an interesting hypothesis to test. This would certainly lead to a better understanding of the (co-)regulation of amino acid metabolism and transport.

### 4.4 Which pathways are responsible for the alleviating effect of Gln on amino acid toxicity?

Because amino acid metabolic pathways are central in cell metabolism, they are the target of several signaling pathways, involving metabolite sensing and hormone responses which are not clearly elucidated yet. Pathway activity is also modulated in response to stresses and changing conditions to provide energy (catabolism during stress) or substrate for protein synthesis (anabolism during permissive conditions) (Pratelli and Pilot, 2014; Hildebrandt, 2018; Heinemann and Hildebrandt, 2021). It has become increasingly clear that the TOR kinase is an intermediate between amino acid sensing and regulation of amino acid anabolism/catabolism (Liu et al., 2021; Ingargiola et al., 2023; Yang et al., 2025), but other cascades have been proposed to also play a role (Gent and Forde, 2017). Interestingly, it has been shown that addition of some amino acids can alter the positive effect of Pro and Ala on nighttime O_2_ consumption rate in a TOR dependent manner (O’leary et al., 2020), reminiscent of the effect of Gln on the toxicity of other amino acids. We tested whether TOR could be involved in the Gln response by using the specific TOR inhibitor AZD8055 on both amino acid uptake and alleviation of amino acid toxicity by Gln. Given that amino acid treatments decrease amino acid uptake (**Figure 7A**), and that amino acids stimulate TOR activity (Cao et al., 2019; O’leary et al., 2020; Liu et al., 2021; O’leary et al., 2025), we expected TOR inhibition to increase amino acid uptake. On the contrary, we observed that AZD8055 led to a decrease in amino acid uptake (**Figure 7B**). Regarding the effect on plant growth, if Gln alleviates Val-triggered growth inhibition through TOR activation, we expected AZD8055 to negate the effect of Gln. Surprisingly, we observed that AZD8055 treatment reduced growth to the same level at all Val concentrations tested, and independent on the presence of Gln (**Figure 6**), which was unexpected if the sole effect of Gln was to activate the TOR pathway. This suggests that the processes affected by Val and Gln lie downstream of TOR, whose overarching control of metabolism masks their individual effects. Whether this hypothesis is compatible with the observation that AZD8055 inhibition of growth could be alleviated by inhibiting Gln synthesis (Ingargiola et al., 2023) remains to be tested. The data are therefore not sufficient to explain the effect of Gln, which could involve the other major player in the regulation of the metabolism, SUCROSE-NON-FERMENTING-1-RELATED PROTEIN KINASE 1 (SnRK1; Artins et al., 2024), or hormonal pathways. A clearer picture would be obtained by either using hypomorphic mutants of the TOR complex (like *raptor1*, Anderson et al., 2005) or measuring TOR activity by assaying the phosphorylation of downstream proteins (like S6K1 or RSP6, Mahfouz et al., 2006; Dobrenel et al., 2016). Unfortunately, those experiments were out of the scope of this manuscript. One could also test the involvement of the putative amino acid sensors of the ACR family (Hsieh and Goodman, 2002; Sung et al., 2011) or the recently identified FYVE1/FREE1 Gln receptor (Tanigawa et al., 2024).

### 4.5 Conclusions and future directions

This work tested and optimized the conditions to study the regulation of amino acid metabolism. Quantifying the growth inhibition of Arabidopsis plants by externally supplied amino acids and its alleviation by Gln revealed that the amount and source of inorganic nitrogen in the medium influence plants’ susceptibility to amino acids and amino acid transport response to external amino acids. Follow-up studies would enable further elucidation of the molecular mechanisms responsible for the growth inhibition by amino acids: the effect of Gln, the interactions between amino acids, the effect of the inorganic nitrogen in the medium, the response of mutants of specific signaling and hormone pathways (TOR, abscisic acid, salicylic acid, jasmonate) to amino acid treatments, and involvement of various hormones could be studied.

## Data Availability

The original contributions presented in the study are included in the article/**Supplementary Material**. Further inquiries can be directed to the corresponding author.

## References

[B1] Anderson G. H. Veit B. Hanson M. R. (2005). The Arabidopsis AtRaptor genes are essential for post-embryonic plant growth. BMC Biol. 3, 12. doi: 10.1186/1741-7007-3-12, PMID: 15845148 PMC1131892

[B2] Artins A. Martins M. C. M. Meyer C. Fernie A. R. Caldana C. (2024). Sensing and regulation of C and N metabolism - novel features and mechanisms of the TOR and SnRK1 signaling pathways. Plant J. 118, 1268–1280. doi: 10.1111/tpj.16684, PMID: 38349940

[B3] Barrada A. Montane M. H. Robaglia C. Menand B. (2015). Spatial regulation of root growth: placing the plant TOR pathway in a developmental perspective. Int. J. Mol. Sci. 16, 19671–19697. doi: 10.3390/ijms160819671, PMID: 26295391 PMC4581319

[B4] Behrend J. Mateles R. I. (1976). Nitrogen metabolism in plant cell suspension cultures: II. Role of organic acids during growth on ammonia. Plant Physiol. 58, 510–512. doi: 10.1104/pp.58.4.510, PMID: 16659706 PMC543259

[B5] Bonner C. A. Jensen R. A. (1997). Recognition of specific patterns of amino acid inhibition of growth in higher plants, uncomplicated by glutamine reversible ‘general amino acid inhibition’. Plant Sci. 130, 133–143. doi: 10.1016/S0168-9452(97)00213-6, PMID: 41777705

[B6] Bonner C. A. Rodrigues A. Miller J. A. Jensen R. A. (1992). Amino acids are general growth inhibitors of *Nicotiana silvestris* in tissue culture. Physiol. Plant 84, 319–328. doi: 10.1111/j.1399-3054.1992.tb04671.x, PMID: 41834780

[B7] Bonner C. A. Williams D. S. Aldrich H. C. Jensen R. A. (1996). Antagonism by L-glutamine of toxicity and growth inhibition caused by other amino acids in suspension cultures of *Nicotiana sylvestris*. Plant Sci. 113, 43–58. doi: 10.1016/0168-9452(95)04284-9

[B8] Borstlap A. C. (1970). Antagonistic effects of branched chain amino acids on the growth of spirodela polyrhiza (L.) schleiden. Acta Bot. Neerl. 19, 211–215. doi: 10.1111/j.1438-8677.1970.tb00643.x, PMID: 41834780

[B9] Borstlap A. C. (1974). Antagonisms between amino acids in the growth of spirodela polyrhiza due to competitive amino acid uptake. Acta Bot. Neerl. 23, 723–738. doi: 10.1111/j.1438-8677.1974.tb00982.x, PMID: 41834780

[B10] Borstlap A. C. (1978). Glycine and L-alanine as antagonists of growth inhibition by the branched-chain amino acids in *spirodela polyrhiza*. J. Exp. Bot. 29, 709–718. doi: 10.1093/jxb/29.3.709

[B11] Borstlap A. C. (1981). Interactions between the branched-chain amino acids in the growth of Spirodela polyrhiza. Planta 151, 314–319. doi: 10.1007/BF00393284, PMID: 24301972

[B12] Cao P. Kim S. J. Xing A. Schenck C. A. Liu L. Jiang N. . (2019). Homeostasis of branched-chain amino acids is critical for the activity of TOR signaling in Arabidopsis. Elife 8, e50747. doi: 10.7554/eLife.50747.sa2, PMID: 31808741 PMC6937141

[B13] Curien G. Bastien O. Robert-Genthon M. Cornish-Bowden A. Cardenas M. L. Dumas R. (2009). Understanding the regulation of aspartate metabolism using a model based on measured kinetic parameters. Mol. Syst. Biol. 5, 271. doi: 10.1038/msb.2009.29, PMID: 19455135 PMC2694679

[B14] Dobrenel T. Mancera-Martinez E. Forzani C. Azzopardi M. Davanture M. Moreau M. . (2016). The arabidopsis TOR kinase specifically regulates the expression of nuclear genes coding for plastidic ribosomal proteins and the phosphorylation of the cytosolic ribosomal protein S6. Front. Plant Sci. 7, 1611. doi: 10.3389/fpls.2016.01611, PMID: 27877176 PMC5100631

[B15] Dunham V. L. Bryan J. K. (1969). Synergistic effects of metabolically related amino acids on the growth of a multicellular plant. Plant Physiol. 44, 1601–1608. doi: 10.1104/pp.44.11.1601, PMID: 16657247 PMC396312

[B16] Farré E. M. Tiessen A. Roessner U. Geigenberger P. Trethewey R. N. Willmitzer L. (2001). Analysis of the compartmentation of glycolytic intermediates, nucleotides, sugars, organic acids, amino acids, and sugar alcohols in potato tubers using a nonaqueous fractionation method. Plant Physiol. 127, 685–700. doi: 10.1104/pp.010280, PMID: 11598242 PMC125103

[B17] Fischer W. N. Loo D. D. Koch W. Ludewig U. Boorer K. J. Tegeder M. . (2002). Low and high affinity amino acid H+-cotransporters for cellular import of neutral and charged amino acids. Plant J. 29, 717–731. doi: 10.1046/j.1365-313X.2002.01248.x, PMID: 12148530

[B18] Foyer C. H. Parry M. Noctor G. (2003). Markers and signals associated with nitrogen assimilation in higher plants. J. Exp. Bot. 54, 585–593. doi: 10.1093/jxb/erg053, PMID: 12508069

[B19] Frechilla S. Lasa B. Aleu M. Juanarena N. Lamsfus C. Aparicio-Tejo P. M. (2002). Short-term ammonium supply stimulates glutamate dehydrogenase activity and alternative pathway respiration in roots of pea plants. J. Plant Physiol. 159, 811–818. doi: 10.1078/0176-1617-00675, PMID: 37640969

[B20] Gamborg O. L. (1970). The effects of amino acids and ammonium on the growth of plant cells in suspension culture. Plant Physiol. 45, 372–375. doi: 10.1104/pp.45.4.372, PMID: 16657321 PMC396416

[B21] Gaufichon L. Rothstein S. J. Suzuki A. (2016). Asparagine metabolic pathways in arabidopsis. Plant Cell Physiol. 57, 675–689. doi: 10.1093/pcp/pcv184, PMID: 26628609

[B22] Gent L. Forde B. G. (2017). How do plants sense their nitrogen status? J. Exp. Bot. 68, 2531–2539. doi: 10.1093/jxb/erx013, PMID: 28201547

[B23] Hacham Y. Schuster G. Amir R. (2006). An *in vivo* internal deletion in the N-terminus region of Arabidopsis cystathionine gamma-synthase results in CGS expression that is insensitive to methionine. Plant J. 45, 955–967. doi: 10.1111/j.1365-313X.2006.02661.x, PMID: 16507086

[B24] Hacham Y. Song L. Schuster G. Amir R. (2007). Lysine enhances methionine content by modulating the expression of S-adenosylmethionine synthase. Plant J. 51, 850–861. doi: 10.1111/j.1365-313X.2007.03184.x, PMID: 17617175

[B25] Hachiya T. Inaba J. Wakazaki M. Sato M. Toyooka K. Miyagi A. . (2021). Excessive ammonium assimilation by plastidic glutamine synthetase causes ammonium toxicity in Arabidopsis thaliana. Nat. Commun. 12, 4944. doi: 10.1038/s41467-021-25238-7, PMID: 34400629 PMC8367978

[B26] Heinemann B. Hildebrandt T. M. (2021). The role of amino acid metabolism in signaling and metabolic adaptation to stress-induced energy deficiency in plants. J. Exp. Bot. 72, 4634–4645. doi: 10.1093/jxb/erab182, PMID: 33993299

[B27] Hildebrandt T. M. (2018). Synthesis versus degradation: directions of amino acid metabolism during Arabidopsis abiotic stress response. Plant Mol. Biol. 98, 121–135. doi: 10.1007/s11103-018-0767-0, PMID: 30143990

[B28] Hirner A. Ladwig F. Stransky H. Okumoto S. Keinath M. Harms A. . (2006). Arabidopsis LHT1 is a high-affinity transporter for cellular amino acid uptake in both root epidermis and leaf mesophyll. Plant Cell 18, 1931–1946. doi: 10.1105/tpc.106.041012, PMID: 16816136 PMC1533986

[B29] Hsieh M. H. Goodman H. M. (2002). Molecular characterization of a novel gene family encoding ACT domain repeat proteins in *Arabidopsis*. Plant Physiol. 130, 1797–1806. doi: 10.1104/pp.007484, PMID: 12481063 PMC166691

[B30] Huang T. Jander G. (2017). Abscisic acid-regulated protein degradation causes osmotic stress-induced accumulation of branched-chain amino acids in Arabidopsis thaliana. Planta 246, 737–747. doi: 10.1007/s00425-017-2727-3, PMID: 28668976

[B31] Husin N. Jalil M. Othman R. Y. Khalid N. (2014). Enhancement of regeneration efficiency in banana (Musa acuminata cv. Berangan) by using proline and glutamine. Sci. Hortic. 168, 33–37. doi: 10.1016/j.scienta.2014.01.013, PMID: 41836151

[B32] Iantcheva A. Zhiponova M. Revalska M. Heyman J. Dincheva I. Badjakov I. . (2022). A common F-box gene regulates the leucine homeostasis of Medicago truncatula and Arabidopsis thaliana. Protoplasma. 259, 277–290. doi: 10.1007/s00709-021-01662-w, PMID: 33973099

[B33] Ingargiola C. Jehanno I. Forzani C. Marmagne A. Broutin J. Clement G. . (2023). The Arabidopsis Target of Rapamycin kinase regulates ammonium assimilation and glutamine metabolism. Plant Physiol. 192, 2943–2957. doi: 10.1093/plphys/kiad216, PMID: 37042394

[B34] Ivanov A. Kameka A. Pajak A. Bruneau L. Beyaert R. Hernandez-Sebastia C. . (2012). Arabidopsis mutants lacking asparaginases develop normally but exhibit enhanced root inhibition by exogenous asparagine. Amino Acids 42, 2307–2318. doi: 10.1007/s00726-011-0973-4, PMID: 21800258

[B35] Janotik A. Dadakova K. Lochman J. Zapletalova M. (2022). L-aspartate and L-glutamine inhibit beta-aminobutyric acid-induced resistance in tomatoes. Plants (Basel) 11, 2908. doi: 10.3390/plants11212908, PMID: 36365361 PMC9655027

[B36] Kan C. C. Chung T. Y. Juo Y. A. Hsieh M. H. (2015). Glutamine rapidly induces the expression of key transcription factor genes involved in nitrogen and stress responses in rice roots. BMC Genomics 16, 731. doi: 10.1186/s12864-015-1892-7, PMID: 26407850 PMC4582844

[B37] Lardos M. Marmagne A. Bonadé Bottino N. Caris Q. Béal B. Chardon F. . (2024). Discovery of the biostimulant effect of asparagine and glutamine on plant growth in Arabidopsis thaliana. Front. Plant Sci. 14. doi: 10.3389/fpls.2023.1281495, PMID: 38317837 PMC10839965

[B38] Lee K. T. Liao H. S. Hsieh M. H. (2023). Glutamine metabolism, sensing, and signaling in plants. Plant Cell Physiol. 64, 1466–1481. doi: 10.1093/pcp/pcad054, PMID: 37243703

[B39] Lee Y. H. Foster J. Chen J. Voll L. M. Weber A. P. Tegeder M. (2007). AAP1 transports uncharged amino acids into roots of Arabidopsis. Plant J. 50, 305–319. doi: 10.1111/j.1365-313X.2007.03045.x, PMID: 17419840

[B40] Less H. Angelovici R. Tzin V. Galili G. (2011). Coordinated gene networks regulating Arabidopsis plant metabolism in response to various stresses and nutritional cues. Plant Cell 23, 1264–1271. doi: 10.1105/tpc.110.082867, PMID: 21487096 PMC3101534

[B41] Less H. Galili G. (2008). Principal transcriptional programs regulating plant amino acid metabolism in response to abiotic stresses. Plant Physiol. 147, 316–330. doi: 10.1104/pp.108.115733, PMID: 18375600 PMC2330312

[B42] Liao H. S. Lee K. T. Chung Y. H. Chen S. Z. Hung Y. J. Hsieh M. H. (2024). Glutamine induces lateral root initiation, stress responses, and disease resistance in Arabidopsis. Plant Physiol. 195, 2289–2308. doi: 10.1093/plphys/kiae144, PMID: 38466723

[B43] Liu Y. Duan X. Zhao X. Ding W. Wang Y. Xiong Y. (2021). Diverse nitrogen signals activate convergent ROP2-TOR signaling in Arabidopsis. Dev. Cell 56, 1283–1295. doi: 10.1016/j.devcel.2021.03.022, PMID: 33831352

[B44] Ma Y. Xu J. Qi J. Zhao D. Jin M. Wang T. . (2024). Crosstalk among plant hormone regulates the root development. Plant Signal. Behav. 19, 2404807. doi: 10.1080/15592324.2024.2404807, PMID: 39279500 PMC11407385

[B45] Mahfouz M. M. Kim S. Delauney A. J. Verma D. P. (2006). Arabidopsis TARGET OF RAPAMYCIN interacts with RAPTOR, which regulates the activity of S6 kinase in response to osmotic stress signals. Plant Cell 18, 477–490. doi: 10.1105/tpc.105.035931, PMID: 16377759 PMC1356553

[B46] Miron D. Ben-Yaacov S. Karchi H. Galili G. (1997). *In vitro* dephosphorylation inhibits the activity of soybean lysine-ketoglutarate reductase in a lysine-regulated manner. Plant J. 12, 1453–1458. doi: 10.1046/j.1365-313x.1997.12061453.x, PMID: 41717205

[B47] Montane M. H. Menand B. (2013). ATP-competitive mTOR kinase inhibitors delay plant growth by triggering early differentiation of meristematic cells but no developmental patterning change. J. Exp. Bot. 64, 4361–4474. doi: 10.1093/jxb/ert242, PMID: 23963679 PMC3808319

[B48] Murashige T. Skoog F. (1962). A revised medium for rapid growth and bio-assays with tobacco tissues culture. Physiol. Plant 15, 473–497. doi: 10.1111/j.1399-3054.1962.tb08052.x, PMID: 41834780

[B49] Noctor G. Novitskaya L. Lea P. J. Foyer C. H. (2002). Co-ordination of leaf minor amino acid contents in crop species: significance and interpretation. J. Exp. Bot. 53, 939–945. doi: 10.1093/jexbot/53.370.939, PMID: 11912236

[B50] O’leary B. M. Honkanen S. Kumari V. Rampitsch C. Nambara E. Millar A. H. (2025). Target of rapamycin signaling in pea embryos is dependent on glutamine but detached from seed storage protein biosynthesis. New Phytol. 248, 2833–2849. doi: 10.1111/nph.70622, PMID: 41097984 PMC12630444

[B51] O’leary B. M. Oh G. G. K. Lee C. P. Millar A. H. (2020). Metabolite regulatory interactions control plant respiratory metabolism via target of rapamycin (TOR) kinase activation. Plant Cell 32, 666–682. doi: 10.1105/tpc.19.00157, PMID: 31888967 PMC7054028

[B52] Pratelli R. Pilot G. (2014). Regulation of amino acid metabolic enzymes and transporters in plants. J. Exp. Bot. 65, 5535–5556. doi: 10.1093/jxb/eru320, PMID: 25114014

[B53] Pratelli R. Voll L. M. Horst R. J. Frommer W. B. Pilot G. (2010). Stimulation of nonselective amino acid export by glutamine dumper proteins. Plant Physiol. 152, 762–773. doi: 10.1104/pp.109.151746, PMID: 20018597 PMC2815850

[B54] Rentsch D. Schmidt S. Tegeder M. (2007). Transporters for uptake and allocation of organic nitrogen compounds in plants. FEBS Lett. 581, 2281–2289. doi: 10.1016/j.febslet.2007.04.013, PMID: 17466985

[B55] Riens B. Lohaus G. Heineke D. Heldt H. W. (1991). Amino acid and sucrose content determined in the cytosolic, chloroplastic, and vacuolar compartments and in the phloem sap of spinach leaves. Plant Physiol. 97, 227–233. doi: 10.1104/pp.97.1.227, PMID: 16668375 PMC1080988

[B56] Stepansky A. Yao Y. Tang G. Galili G. (2005). Regulation of lysine catabolism in Arabidopsis through concertedly regulated synthesis of the two distinct gene products of the composite AtLKR/SDH locus. J. Exp. Bot. 56, 525–536. doi: 10.1093/jxb/eri031, PMID: 15569707

[B57] Sung T. Y. Chung T. Y. Hsu C. P. Hsieh M. H. (2011). The ACR11 encodes a novel type of chloroplastic ACT domain repeat protein that is coordinately expressed with GLN2 in Arabidopsis. BMC Plant Biol. 11, 118. doi: 10.1186/1471-2229-11-118, PMID: 21861936 PMC3173338

[B58] Svietlova N. Zhyr L. Reichelt M. Grabe V. Mithofer A. (2024). Glutamine as sole nitrogen source prevents induction of nitrate transporter gene NRT2.4 and affects amino acid metabolism in Arabidopsis. Front. Plant Sci. 15, 1369543. doi: 10.3389/fpls.2024.1369543, PMID: 38633457 PMC11022244

[B59] Tanigawa M. Maeda T. Isono E. (2024). FYVE1/FREE1 is involved in glutamine-responsive TORC1 activation in plants. iScience 27, 110814. doi: 10.1016/j.isci.2024.110814, PMID: 39297172 PMC11409180

[B60] Vasudevan A. Selvaraj N. Ganapathi A. Kasthurirengan S. Ramesh Anbazhagan V. Manickavasagam M. (2004). Glutamine: A suitable nitrogen source for enhanced shoot multiplication in cucumis sativus L. Biol. Plantarum 48, 125–128. doi: 10.1023/B:BIOP.0000024288.82679.50, PMID: 38124636

[B61] Winter H. C. Lohaus G. Heldt H. W. (1992). Phloem transport of amino acids in relation to their cytosolic levels in barley leaves. Plant Physiol. 99, 996–1004. doi: 10.1104/pp.99.3.996, PMID: 16669030 PMC1080575

[B62] Winter H. Robinson D. G. Heldt H. W. (1993). Subcellular volumes and metabolite concentrations in barley leaves. Planta 191, 180–190. doi: 10.1007/BF00199748, PMID: 41836790

[B63] Winter H. Robinson D. G. Heldt H. W. (1994). Subcellular volumes and metabolite concentrations in spinach leaves. Planta 193, 530–535. doi: 10.1007/BF02411558, PMID: 41836790

[B64] Wu C. C. Singh P. Chen M. C. Zimmerli L. (2010). L-Glutamine inhibits beta-aminobutyric acid-induced stress resistance and priming in Arabidopsis. J. Exp. Bot. 61, 995–1002. doi: 10.1093/jxb/erp363, PMID: 20007686 PMC2826644

[B65] Xu H. Wang F. Rebecca Njeri D. Chen X. Lin Z. (2024). Molecular mechanisms underlying the signal perception and transduction during seed germination. Mol. Breed. 44, 27. doi: 10.1007/s11032-024-01465-w, PMID: 38525006 PMC10954596

[B66] Yang L. Zhang R. Zhang H. Yang Y. Fu L. (2025). TOR mediates stress responses through global regulation of metabolome in plants. Int. J. Mol. Sci. 26, 2095. doi: 10.3390/ijms26052095, PMID: 40076716 PMC11900525

[B67] Zhao J. He Y. Zhang H. Wang Z. (2024). Advances in the molecular regulation of seed germination in plants. Seed Biol. 3, 0–0. doi: 10.48130/seedbio-0024-0011

[B68] Zhu X. Tang G. Galili G. (2002). The activity of the Arabidopsis bifunctional lysine-ketoglutarate reductase/saccharopine dehydrogenase enzyme of lysine catabolism is regulated by functional interaction between its two enzyme domains. J. Biol. Chem. 277, 49655–49661. doi: 10.1074/jbc.M205466200, PMID: 12393892

[B69] Zhu-Shimoni J. X. Galili G. (1998). Expression of an arabidopsis aspartate Kinase/Homoserine dehydrogenase gene is metabolically regulated by photosynthesis-related signals but not by nitrogenous compounds. Plant Physiol. 116, 1023–1028. doi: 10.1104/pp.116.3.1023, PMID: 9501134 PMC35071

