# Supplementary material for: Glutamine alleviates the toxicity of externally applied amino acids in Arabidopsis
Source: Front Plant Sci. 2026 Mar 19;17:1689741. doi: 10.3389/fpls.2026.1689741 (PMC13044093; doi:10.3389/fpls.2026.1689741)
Supplement: Supplementary file 1 [file DataSheet1.pdf]

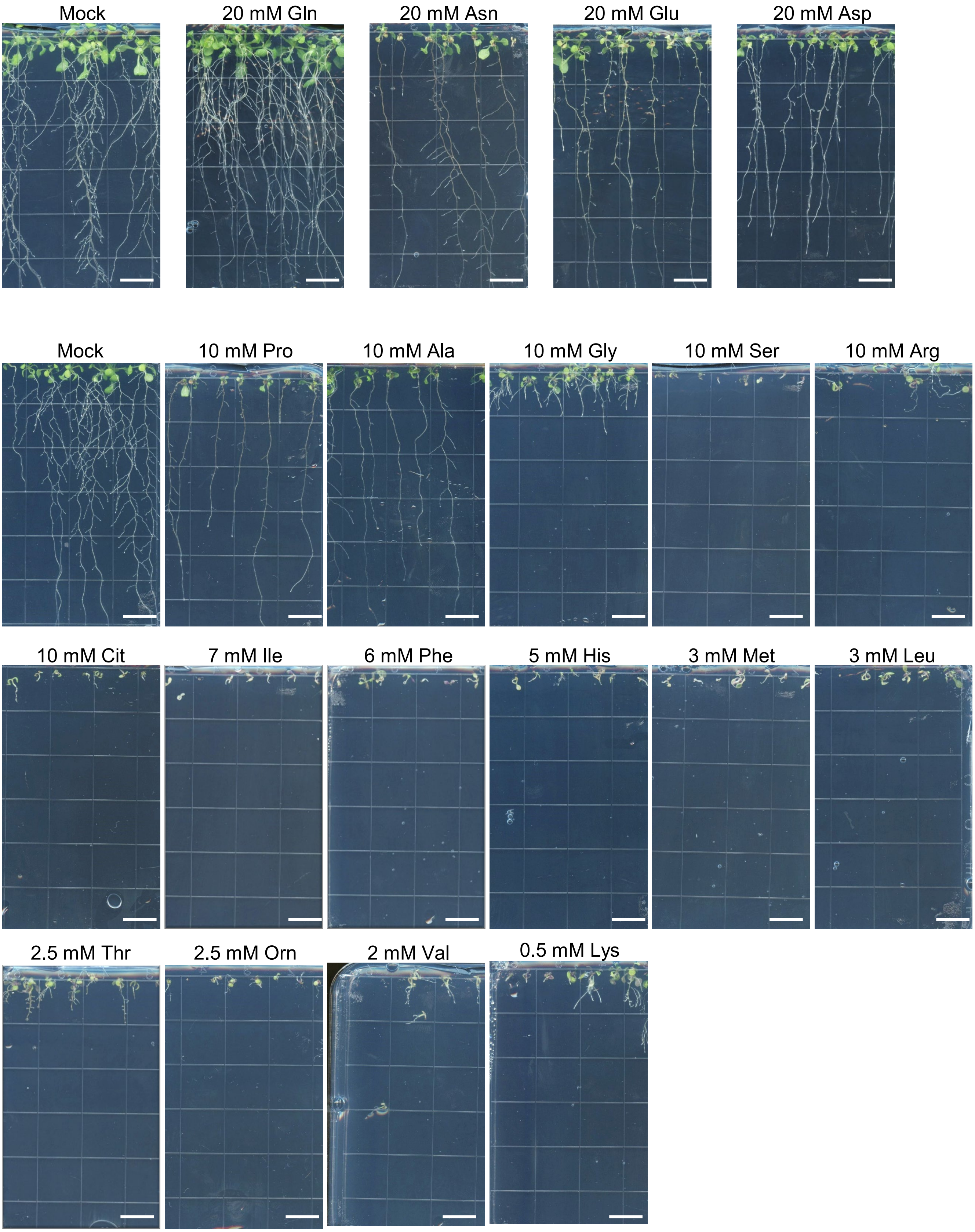

Supplementary Figure 1

Plants were grown vertically for 14 days on base medium containing 5 mM KNO_3 and amino acids at the indicated concentrations. The top row and the remaining rows of pictures correspond to two different experiments, each with their respective mock treatment. Scale bars = 1 cm.

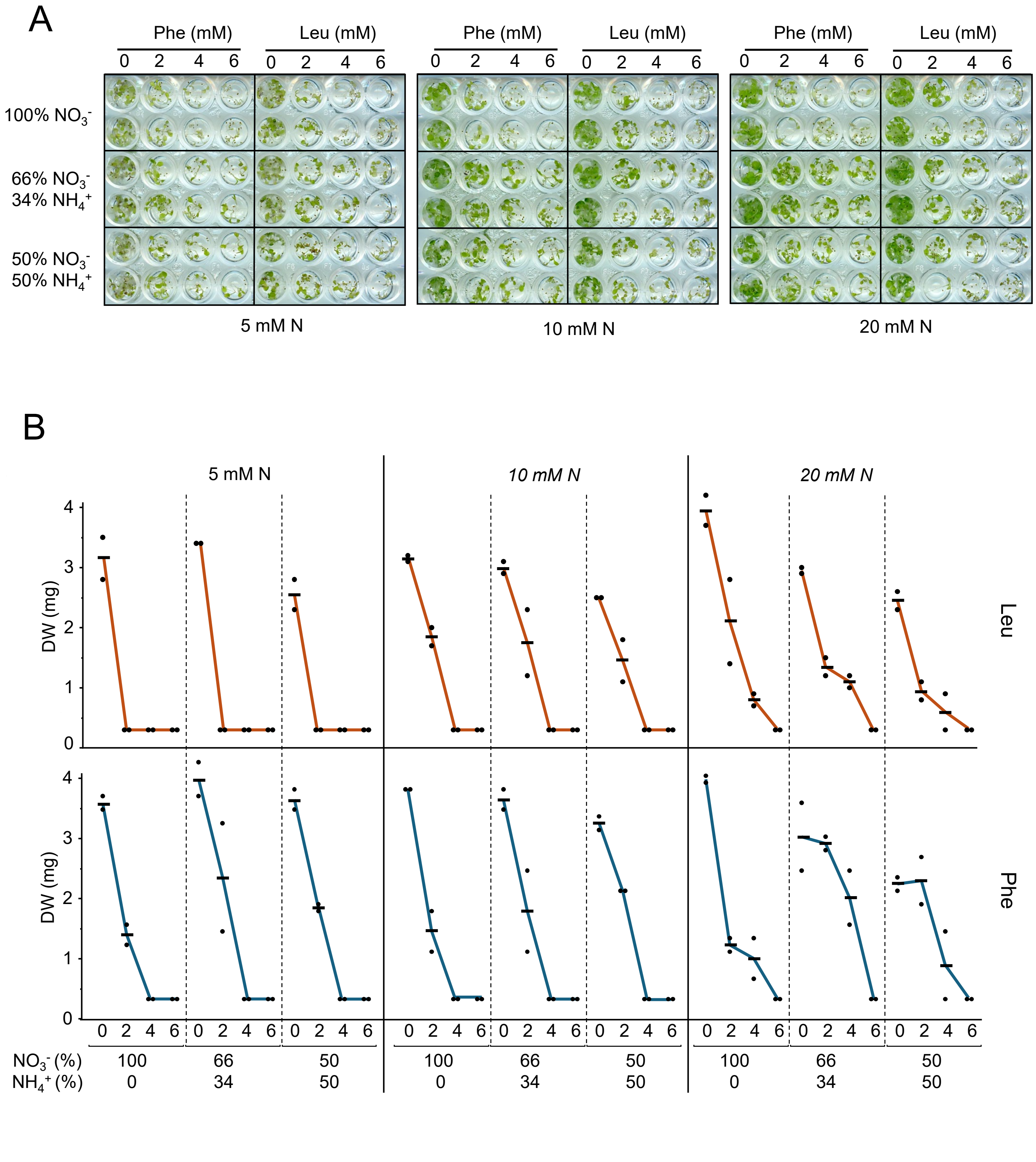

Supplementary Figure 2

Arabidopsis plants were grown in the presence of Leu or Phe and various inorganic nitrogen sources and concentrations. After germination, plants were grown for 9 days in base medium containing from 5 to 10 mM nitrogen, brought as 100, 66 or 50% nitrate and 0, 33 or 50% ammonium, respectively, and 0, 2, 4 or 6 mM Phe or Leu. **A.** Picture of the plants at the end of the experiment. **B.** Dry weight of the plants, indicated by dots; horizontal bars represent the average (n=2).

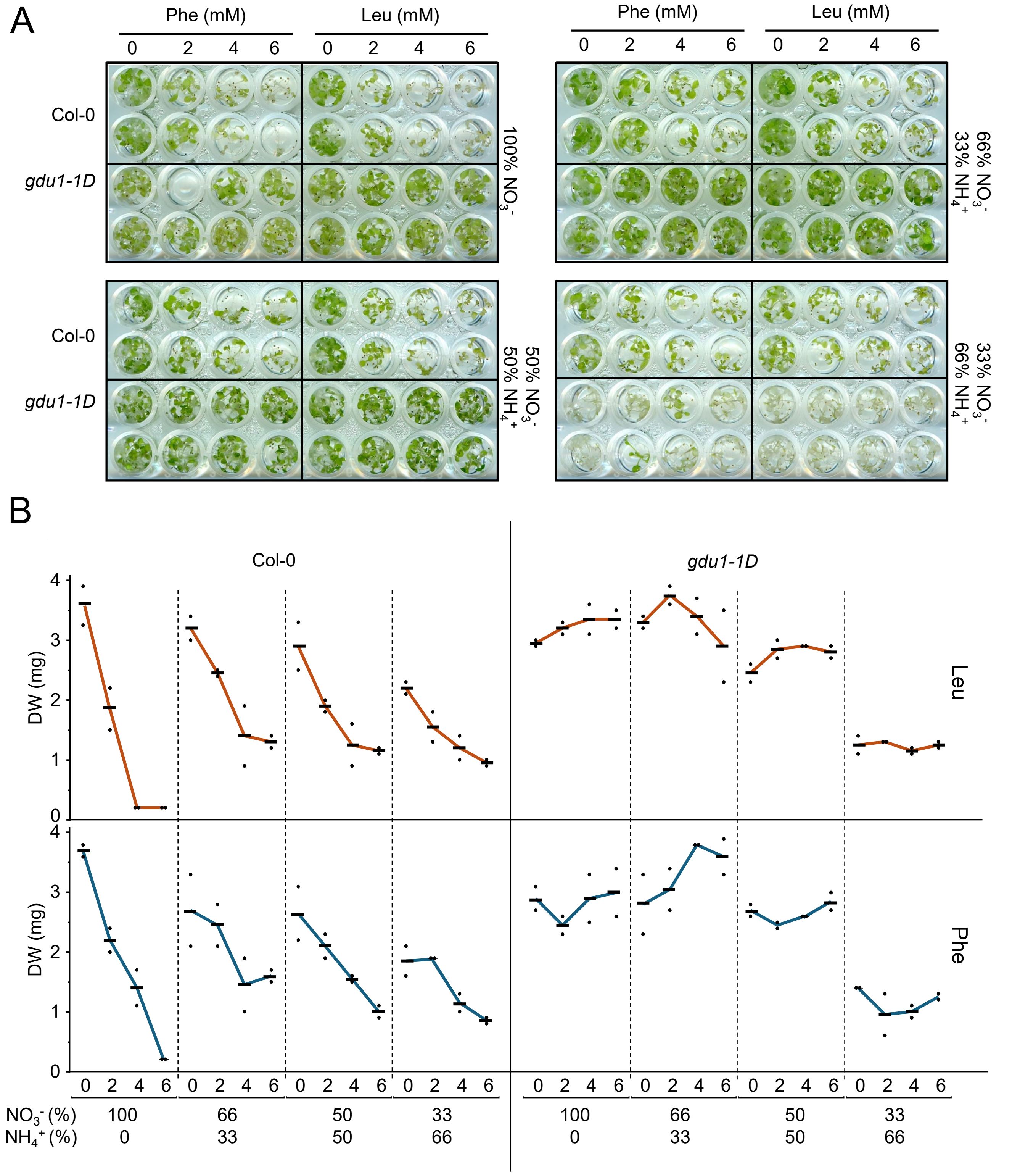

Supplementary Figure 3

Wild type and *gdu1-1D* Arabidopsis plants were grown in the presence of Leu or Phe and various inorganic nitrogen sources. After germination, plants were grown for 9 days in base medium containing 10 mM nitrogen, brought as 100, 66. 50 or 33% nitrate and 0, 33, 50 or 66% ammonium, respectively, and 0, 2, 4 or 6 mM Phe or Leu. **A.** Picture of the plants at the end of the experiment. **B.** Dry weight of the plants, indicated by dots; horizontal bars represent the average (n=2).

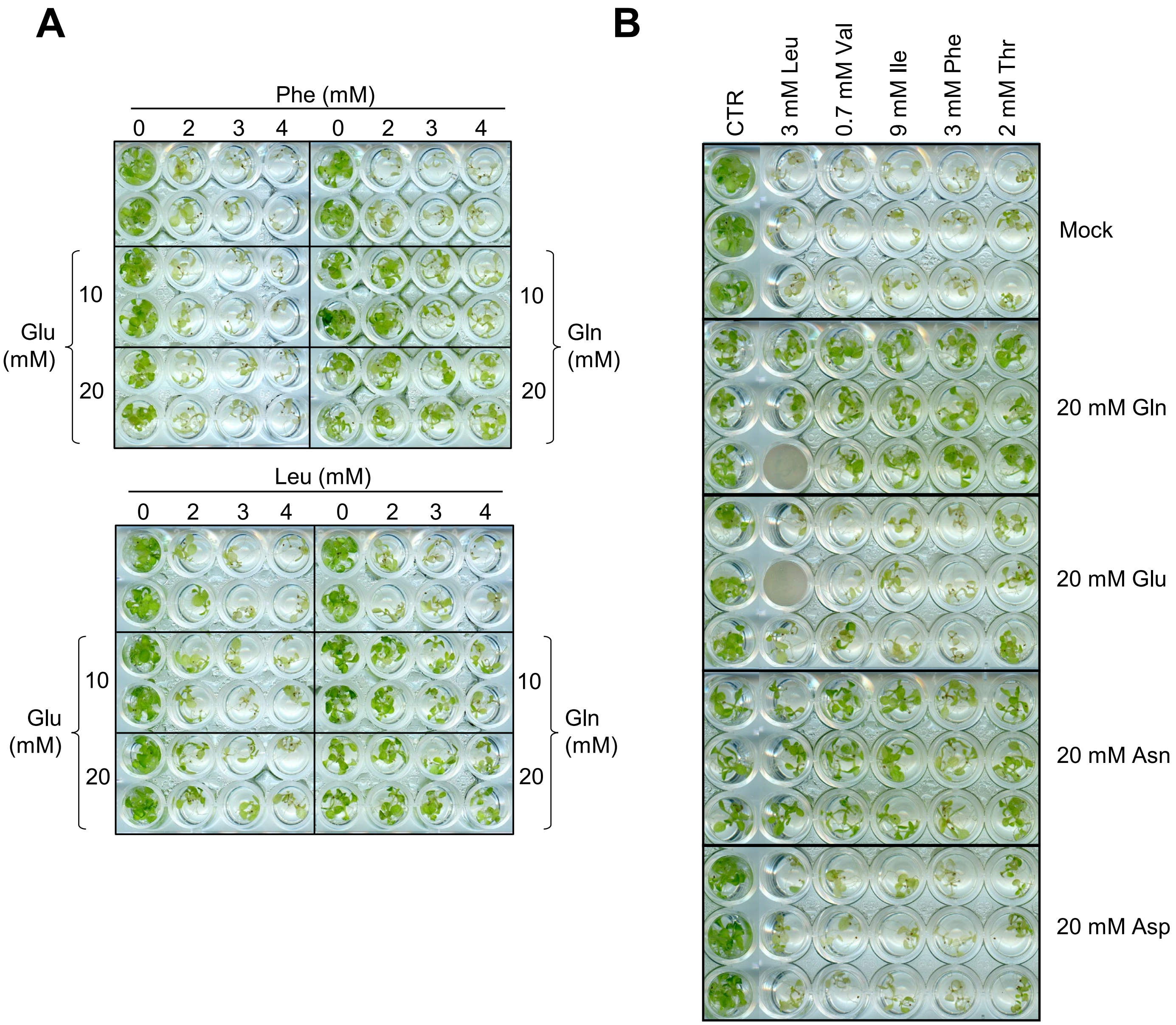

Supplementary Figure 4

A. Pictures of Arabidopsis plants germinated for 4 days on solid medium and grown for 9 days in liquid base medium containing 10 mM KNO_3, 0, 2, 3 or 4 mM Phe or Leu, and 10 or 20 mM Gln or Glu, as indicated.

B. Pictures of Arabidopsis plants germinated for 4 days on solid medium and grown for 9 days in liquid base medium containing 10 mM KNO_3, supplemented with Leu, Val, Ile, Phe and Thr at the indicated concentrations and 20 mM Gln or Glu, as indicated.

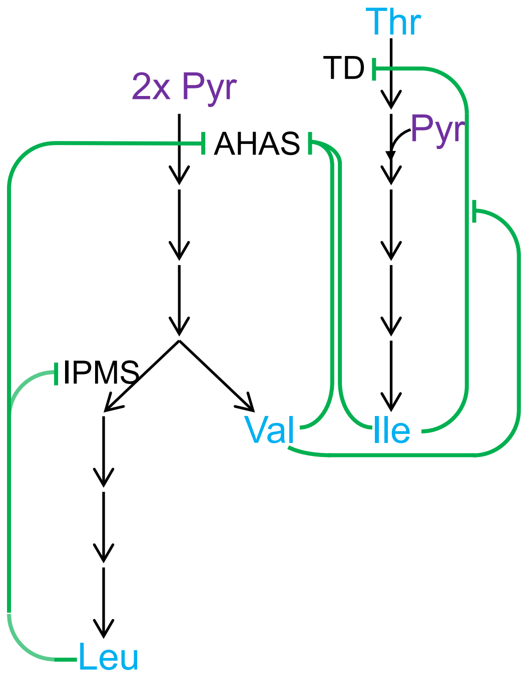

Supplementary Figure 5

Overview of the BCAA biosynthesis pathways with known feedback regulation of the metabolic enzymes. TD: Thr deaminase, AHAS: Acetohydroxyacid acid synthase, IMPS: Isopropylmalate synthase, Pyr: pyruvate.

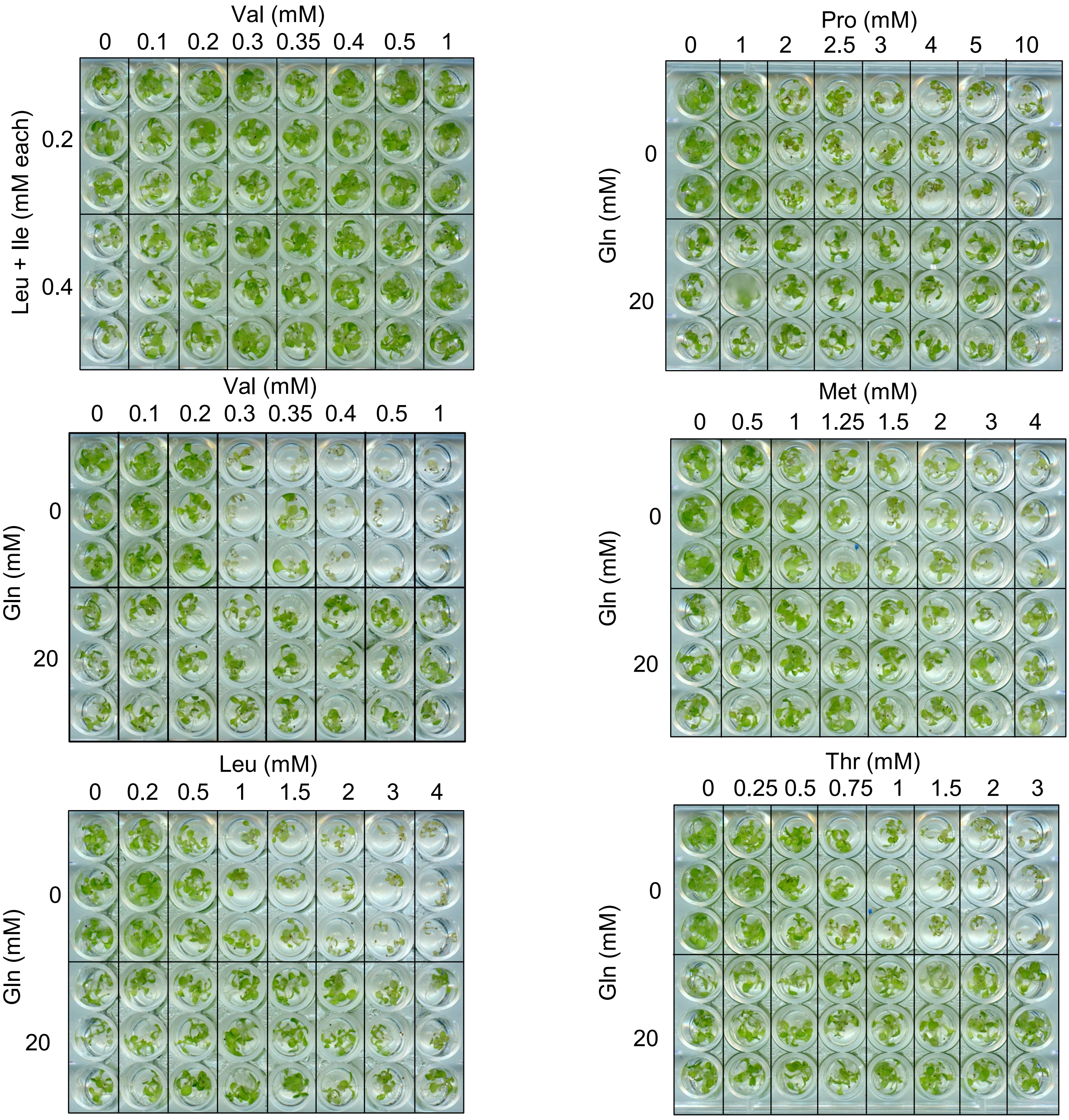

Supplementary Figure 6

Picture of the plants leading to results from Figure 4. See Figure 4 legend for details.

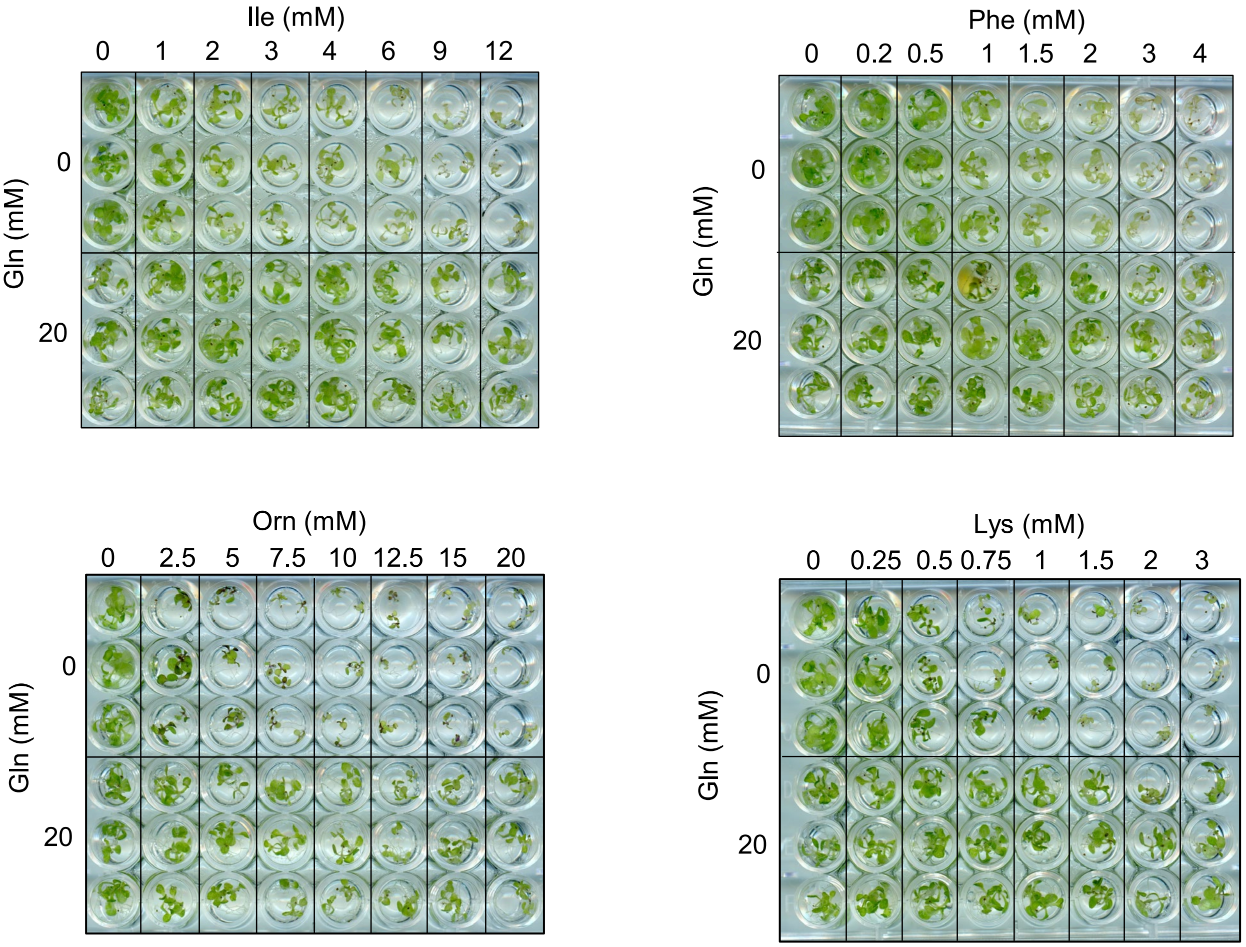

Supplementary Figure 6 (continued)
Picture of the plants leading to results from Figure 4. See Figure 4 legend for details.

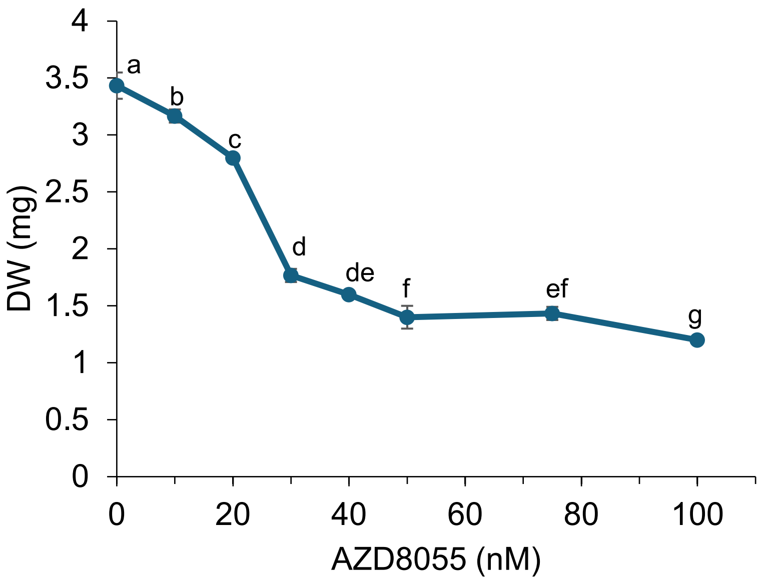

Supplementary Figure 7
Dose response curves of Arabidopsis plant dry weight for AZD8055. After four days of germination on solid medium, plants were grown for 9 days in the base medium containing 10 mM KNO3, supplemented AZD8055 at the indicated concentrations (X-axis). The effect of concentration on the dry weight was analyzed using a one-way ANOVA followed by a Tukey's HSD, P-value <0.05. Error bars (when visible) represent the standard deviation from the mean, n=3. Different letters represent statistically different means.
